# Prioritized learning of cross-population neural dynamics

**DOI:** 10.1088/1741-2552/ade569

**Published:** 2025-08-11

**Authors:** Trisha Jha, Omid G Sani, Bijan Pesaran, Maryam M Shanechi

**Affiliations:** 1 Ming Hsieh Department of Electrical and Computer Engineering, Viterbi School of Engineering, University of Southern California, Los Angeles, CA, United States of America; 2 Departments of Neurosurgery, Neuroscience, and Bioengineering, University of Pennsylvania, Philadelphia, PA, United States of America; 3 Thomas Lord Department of Computer Science, Alfred E. Mann Department of Biomedical Engineering, Neuroscience Graduate Program, University of Southern California, Los Angeles, CA, United States of America

**Keywords:** multiregional modeling, linear dynamical systems, dimensionality reduction

## Abstract

*Objective*. Improvements in recording technology for multi-region simultaneous recordings enable the study of interactions among distinct brain regions. However, a major computational challenge in studying cross-regional, or cross-population dynamics in general, is that the cross-population dynamics can be confounded or masked by within-population dynamics. *Approach*. Here, we propose cross-population prioritized linear dynamical modeling (CroP-LDM) to tackle this challenge. CroP-LDM learns the cross-population dynamics in terms of a set of latent states using a prioritized learning approach, such that they are not confounded by within-population dynamics. Further, CroP-LDM can infer the latent states both causally in time using only past neural activity and non-causally in time, unlike some prior dynamic methods whose inference is non-causal. *Main results*. First, through comparisons with various LDM methods, we show that the prioritized learning objective in CroP-LDM is key for accurate learning of cross-population dynamics. Second, using multi-regional bilateral motor and premotor cortical recordings during a naturalistic movement task, we demonstrate that CroP-LDM better learns cross-population dynamics compared to recent static and dynamic methods, even when using a low dimensionality. Finally, we demonstrate how CroP-LDM can quantify dominant interaction pathways across brain regions in an interpretable manner. *Significance*. Overall, these results show that our approach can be a useful framework for addressing challenges associated with modeling dynamics across brain regions.

## 1. Introduction

Mapping out the interaction pathways among different brain regions is a challenging problem in neuroscience since tasks carried out by the brain rely on coordination among several distinct regions (Heekeren *et al* 2008, Pinto *et al* 2019, Steinmetz *et al* 2019). Having the ability to model and quantify the strength of interactions between brain regions can help deepen our understanding of how the brain carries out these tasks. Improvements in recording technology as well as the availability of multi-area simultaneous recordings now make it possible to study interactions across multiple brain regions (Jun *et al* 2017, Yang and Yuste 2017, Dimwamwa *et al* 2024).

Much of the prior work studying interactions between brain regions, or more generally between neural populations, has employed static methods. That is, these methods do not explicitly consider the temporal structure of the data. One line of recent studies (Kaufman *et al* 2014, Perich *et al* 2018, Ruff and Cohen 2019) have used common static dimensionality reduction techniques such as principal component regression and factor regression to study interactions between brain regions. These approaches first describe the activity in one region in terms of a low dimensional latent variable, before relating that latent variable to the activity in a second region. As such, this class of static approaches extract latent variables that describe the activity in one region without taking into consideration the activity in the other region. In contrast, another line of studies develop other static dimensionality reduction methods such as reduced rank regression (Izenman 1975) (RRR), canonical correlation analysis (Hotelling 1936), and partial least squares (Wold 1975), which learn latent variables that are shared between the two brain regions by performing dimensionality reduction using activity from both brain regions. Many studies (Semedo *et al* 2019, Veuthey *et al* 2020, Srinath *et al* 2021) have used these static methods to describe population interactions across regions. These static methods, however, may not explain neural variability as accurately as dynamical methods that explicitly consider and model the temporal nature of the neural time-series data.

Recent works have started to consider how population interactions evolve across time. One approach used in these works is to apply static methods in sliding windows across time (Rodu *et al* 2018, Semedo *et al* 2021). Other approaches estimate the directionality and quantify the lead-lag relationship across neural populations via descriptive models (Adhikari *et al* 2010, Jiang *et al* 2015, Rodu *et al* 2018) but they still do not provide generative dynamical models of these interactions. Other works (Glaser *et al* 2020, Gokcen *et al* 2022) have made progress in explicitly accounting for dynamics when modeling interactions across brain regions by using dynamic models to simultaneously describe the activity of multiple regions.

Despite all these advances, a major outstanding challenge is that the shared dynamics across two regions may be masked by, mistaken for, or confounded by within-region dynamics. We define shared dynamics as dynamics in one region that are predictive of dynamics in another region, potentially reflecting interaction across the regions. This issue occurs because these prior methods jointly maximize the data log-likelihood of both the shared and within-region activity. Another challenge is that several of these prior approaches do not provide a method for extracting shared dynamics using only ‘past’ neural data from the regions, i.e. causally in time; this limits the temporal interpretability of shared dynamics. In this work, we address these challenges by prioritizing the learning of shared cross-population dynamics over within-population dynamics. We also allow extraction of cross-population dynamics exclusively using past neural data, while also enabling extraction using past and future data if desired. We do so by introducing cross-population prioritized linear dynamical modeling (CroP-LDM), a new formulation for prioritizing the learning of dynamics that are shared across neural populations. Since our main goal is to provide a tool for investigating neural interactions within and across brain regions, we focus on linear modeling because it provides a simple, interpretable description of interactions while still maintaining reasonable expressiveness.

Given neural activity from two neural populations, CroP-LDM learns a dynamical model that prioritizes the extraction of cross-population dynamics over within-population dynamics; this is done by setting the learning objective to be accurate prediction of the target neural population activity from the source neural population activity, i.e. cross-population prediction. This explicit prioritization can help learn the cross-population dynamics more accurately as we will show. Also, the objective is designed to explicitly dissociate cross- and within-population dynamics, thus ensuring that the extracted dynamics correspond to cross-population interactions alone and are not mixed with within-population dynamics. While various analytical or numerical techniques can be used to optimize this objective in CroP-LDM, to enable learning efficiency, we take a subspace identification learning approach similar to preferential subspace identification (Sani *et al* 2021).

In addition to supporting prioritized learning, the CroP-LDM framework supports inference of dynamics both causally in time using only past neural data at each time-step (filtering), and non-causally in time using all data at each time-step (smoothing). This is unlike prior methods for modeling cross-regional dynamics, which only support one or the other. This is also unlike (Sani *et al* 2021) that supports causal filtering alone for modeling behaviorally relevant neural dynamics. Because in causal filtering all model predictions are functions of past input data, this can aid interpretability, as it ensures that information predicted in the target region appeared first in the source region. Non-causal smoothing loses this interpretation, as both past and future source data are used to predict the target region. Nevertheless, non-causal smoothing can more accurately infer latent dynamics/states due to the use of future information. As such, non-causal smoothing may be desired in some applications, especially when working with noisy neural data. Thus, we develop CroP-LDM as a versatile tool that flexibly allows for both causal filtering or non-causal smoothing, based on data quality and specific analysis goals.

Finally, another challenge in interpreting cross-population dynamics is that, even if population A is predictive of population B, this predictive information may already exist in population B itself. To address this challenge in interpretation, we further incorporate a partial ${R^2}$ metric to quantify the *non-redundant* information that one population provides about another.

We extensively validate CroP-LDM in multiple analyses. To demonstrate that the prioritization enabled by our learning procedure is important for accurate modeling of cross-population dynamics, we compare to two other linear dynamical system-based models. First, we formulate an approach that fits the same model structure as CroP-LDM by numerically optimizing the joint log-likelihood of both cross and within-population dynamics, without prioritizing the cross-population dynamics. Second, we compare with non-prioritized LDM, which first fits an LDM to the source population activity and then regresses the states to the target activity. We establish in simulations that CroP-LDM achieves more accurate and efficient learning of cross-population dynamics compared to these alternative linear dynamical system-based models.

Next, we evaluate CroP-LDM for modeling of cross- and within-region dynamics on multiregional motor and premotor cortical activity from non-human primates (NHP) and compare it with recently developed static and dynamic methods designed to model cross-regional interactions (Semedo *et al* 2019, Gokcen *et al* 2022). To model cross-region dynamics, we take a neural population from each region and model their shared dynamics with CroP-LDM. To model within-region dynamics, we take two non-overlapping populations within that region and again use CroP-LDM to model the dynamics shared across these two populations within that single region. We first find that CroP-LDM extracts the cross-population dynamics more accurately than these recent methods (Semedo *et al* 2019, Gokcen *et al* 2022). We then find that, with its prioritized approach to learning, CroP-LDM can represent the cross-region and within-region dynamics using lower dimensional latent states than the prior dynamic method (Gokcen *et al* 2022).

Finally, we demonstrate that our approach can identify and quantify dominant interaction pathways between brain regions. In our first dataset consisting of premotor and motor cortex areas, CroP-LDM quantifies that PMd can better explain M1 than vice versa, consistent with prior biological evidence. In our second dataset, which consists of bilateral recordings from a monkey performing a task with its right hand, we find that interactions within the left hemisphere were dominant, again showing that CroP-LDM can lead to biologically-consistent interpretations. Overall, these results suggest that CroP-LDM can be a useful tool for investigating shared cross-population dynamics with priority such that they are not masked by, mistaken for, or confounded by within-population dynamics.

## 2. Methods

### 2.1. Neural recordings, behavioral task, and data processing

The neural data analyzed in this study come from the motor cortical regions of two monkeys performing 3D reach, grasp, and return movements to diverse locations with their right arm. All surgical and experimental procedures were performed in compliance with the National Institute of Health Guide for Care and Use of Laboratory Animals and were approved by the New York University Institutional Animal Care and Use Committee. For Monkey J, an electrode array with 137 electrodes was used to record from the left hemisphere of the brain in regions M1, PMd, PMv, and PFC, with 28, 32, 45, and 32 electrodes in each area, respectively. For Monkey C, four 32-electrodes microdrives were used to record from PMd and PMv on both the left and right hemispheres. The behavioral data consists of the angular position of 27 (Monkey J) or 25 (Monkey C) joints of the upper-extremity (Abbaspourazad *et al* 2021, Sani *et al* 2021).

In our analysis, we use non-smoothed spike counts in non-overlapping 50 ms bins as our neural activity. We use the joint angles at the end of each 50 ms timestep of neural activity as our behavioral activity. Data consisted of 7 recording sessions for Monkey J and 4 recording sessions for Monkey C.

### 2.2. CroP-LDM algorithm

#### 2.2.1. Model formulation

We present the CroP-LDM algorithm and the methodology for applying this algorithm to study interactions across neural populations. Our model formulation is as follows:

\begin{align*}&amp;\left\{ {\begin{array}{@{}lc} {\begin{array}{@{}l} {{x_{k + 1}} = A{x_k} + {w_k}} \\ {{a_k} = {C_a}{x_k} + {v_k}} \\ {{b_k} = {C_b}{x_k} + { \epsilon _k}} \end{array},{x_k} = \left[ {\begin{array}{*{20}{l}} {x_k^{\left( 1 \right)}} \\ {x_k^{\left( 2 \right)}} \end{array}} \right]{\text{, }}} \\ \end{array}} \right.\nonumber\\ &amp;\quad{{C_a} = \left[ \!{\begin{array}{@{}cc@{}} {{C_{{a_1}}}}&amp;{{C_{{a_2}}}} \end{array}} \!\right],{C_b} = \left[{\begin{array}{@{}cc@{}} {{C_{{b_1}}}}&amp;0 \end{array}} \right]}. \end{align*}

**Figure 1.**
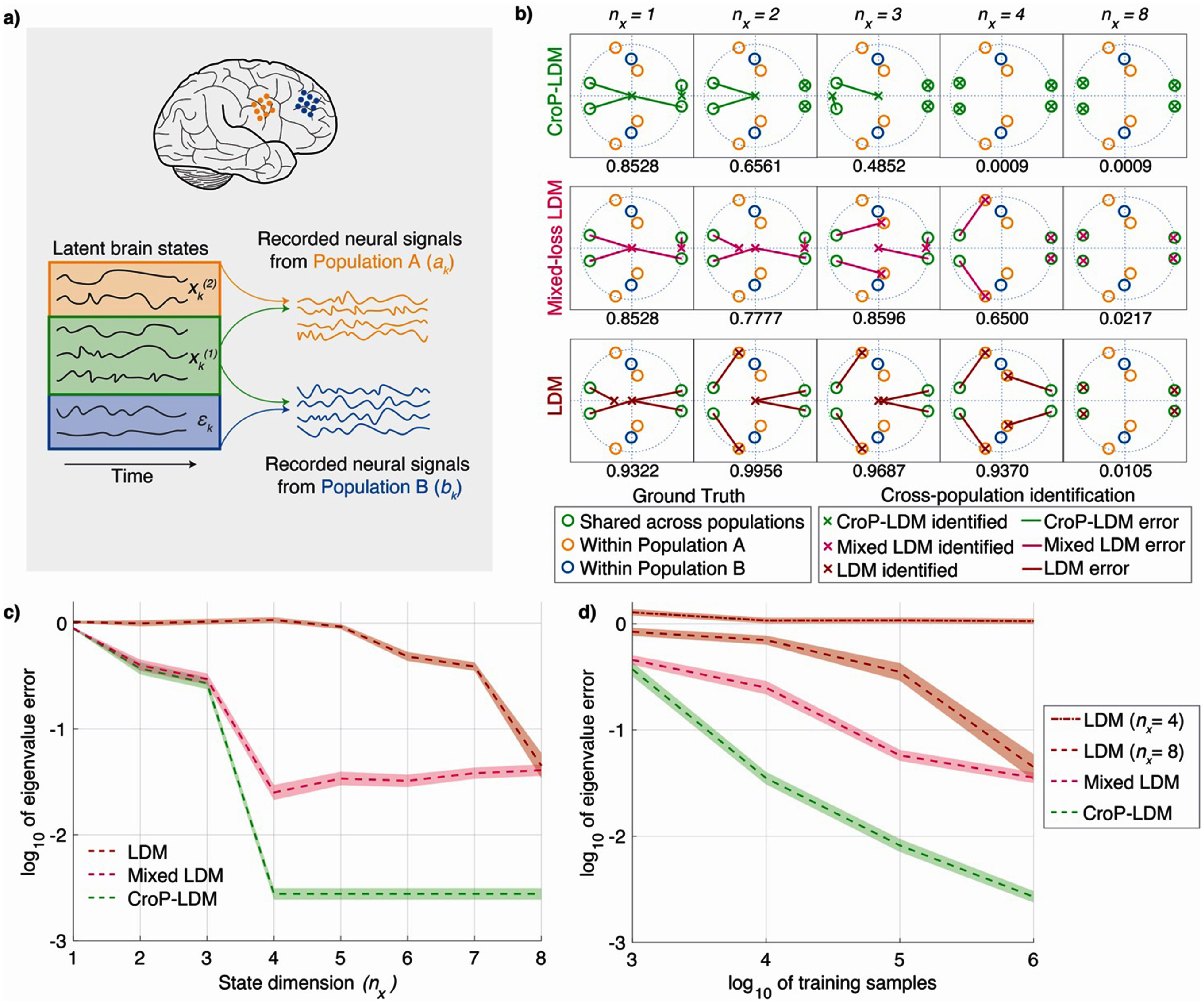
CroP-LDM enables learning of cross-population dynamics. (a) The high-dimensional latent state of the brain, ${x_k},{\text{ }}$ can be decomposed into several components. Dimensions $x_k^{\left( 1 \right)}$ and $x_k^{\left( 2 \right)}$ drive neural activity in Population A while dimensions $x_k^{\left( 1 \right)}$ and ${ \epsilon _k}$ drive neural activity in Population B. Since $x_k^{\left( 1 \right)}$ drives activity in both populations, it represents the cross-population shared dynamics. (b) We present a case study from one of our simulated datasets. We show the true and identified shared eigenvalues for CroP-LDM (top), mixed-loss LDM (middle), and non-prioritized LDM (bottom) for different latent state dimensions. The eigenvalues are shown on the complex plane, and the unit circle is shown with the dotted blue line. The true eigenvalues are marked with colored circles and identified eigenvalues are marked with colored crosses. When the total state dimension ${n_x}$ is less than the shared cross-population state dimension $({n_1} = 4)$, we take the remaining ${n_1} - {\text{ }}{n_x}$ eigenvalues to be 0. (c) Normalized eigenvalue error as a function of total state dimension is shown for LDM, mixed-loss LDM, and CroP-LDM. Dotted lines show the average error, and shaded areas indicate the s.e.m. ($n = 50$ random models). (d) Normalized eigenvalue error as a function of number of training samples is shown for LDM of different state dimensions, mixed-loss LDM at ${n_x} = 8$, and CroP-LDM at ${n_x} = 4$. Dotted lines show the average error, and shaded areas indicate the s.e.m. ($n = 50$ random models).

Here, ${a_k} \in {\mathbb{R}^{{n_a}}}$ is the recorded neural activity in population A and ${b_k} \in {\mathbb{R}^{{n_b}}}$ is the recorded neural activity in population B (figure 1(a)). The latent state ${x_k} \in {\mathbb{R}^{{n_x}}}$, composed of sections $x_k^{\left( 1 \right)} \in {\mathbb{R}^{{n_1}}}$ and $x_k^{\left( 2 \right)} \in {\mathbb{R}^{{n_2}}}$ (with ${n_2} = {n_x} - {n_1}$), models the recorded neural activity in population A. Specifically, $x_k^{\left( 1 \right)}$ models the dynamics of population A shared with population B and $x_k^{\left( 2 \right)}$ models the within-population A dynamics not shared with population B. ${w_k}$ and ${v_k}$ are zero-mean white noise processes independent of the latent state with the following cross-correlations:

\begin{equation*}{ }E\left\{ {\left[ {\begin{array}{*{20}{c}} {{w_k}} \\ {{v_k}} \end{array}} \right]\left[ {\begin{array}{*{20}{c}} {w_k^{\mathrm{T}}}&amp;{v_k^{\mathrm{T}}} \end{array}} \right]} \right\} \triangleq \left[ {\begin{array}{*{20}{c}} Q&amp;S \\ {{S^{\mathrm{T}}}}&amp;R \end{array}} \right].\end{equation*}

Additionally, ${\epsilon _k} \in {\mathbb{R}^{{n_b}}}{\text{ }}$ represents independent dynamics within population B that are not described by ${x_k}$. So, only the $x_k^{\left( 1 \right)}$ components of the latent state relate to activity in population B. The remaining within-population B dynamics are represented by ${ \epsilon _k}$, which is assumed to be non-white, zero-mean and independent of latent state ${x_k}$ and noises ${w_k}$ and ${v_k}$. With this assumption and based on equation (1), ${ \epsilon_k}$ will also be independent of ${a_k}$, which means the observed activity in population A (i.e. ${a_k}$) does not provide any information about the within-population B dynamics (i.e. ${\epsilon _k}$) other than the shared ones (i.e. $x_k^{\left( 1 \right)})$. Our primary goal is to learn $x_k^{\left( 1 \right)}$ and the associated parameters for cross-population interactions and do so in a manner that prioritizes their learning. We will next describe how CroP-LDM learns the parameters of this model.

#### 2.2.2. Learning the model parameters

To learn the model parameters from training data, we use a method that can directly learn the parameters related to the $x_k^{\left( 1 \right)}$ states from equation (1) without learning the other model parameters (see below). We do so because this allows us to fit models with low dimensional latent states, where the total state dimension is the dimension of $x_k^{\left( 1 \right)}$ rather than that of ${\text{ }}{x_k}$. By design, this learning approach ensures that the latent states learned by the model (i.e. $x_k^{\left( 1 \right)}$ only) capture the dynamics in ${a_k}$ shared with ${b_k}$, i.e. cross-population dynamics.

Here, we can use this same approach to study both cross-region and within-region neural interactions by taking ${a_k}$ and ${b_k}$ to be non-overlapping populations either in two different regions or in the same region, respectively (details in section 2.6). This unified approach eliminates the need for learning $x_k^{\left( 2 \right)}$ and its associated model parameters.

Given training neural activity ${a_k}$ and ${b_k}$ from both regions, CroP-LDM learns the cross-population latent states $x_k^{\left( 1 \right)}$ and associated parameters. To do so, we adopt algebraic operations similar to those developed as the ‘first stage’ in (Sani *et al* 2021). Specifically, CroP-LDM first estimates the latent states by projecting future neural activity from ${b_k}$ onto past neural activity from ${a_k}$. Given the extracted latent states, the associated model parameters are learned via least squares regression (see also appendix A for an expanded block structured formulation of the model and how parameters related to $x_k^{\left( 2 \right)}$ can be learned if desired, though this is not our focus). Once model parameters are learned, we can infer the latent states $x_k^{\left( 1 \right)}$ either with causal filtering or with non-causal smoothing in the test set as explained next. This is unlike prior work on multi-regional modeling that can do one or the other.

#### 2.2.3. Causal CroP-LDM to enable causal state filtering

One main advantage of CroP-LDM compared with prior models of cross-region interactions is the flexible ability to extract the latent states both causally in time using just past data from a neural population for ease of interpretation, or non-causally in time using all data from a neural population for better state estimation.

For causal filtering, once the model is learned using the training data, we can use a Kalman predictor in the test data to extract the relevant states at each time $k + 1$ given population A’s past neural activity ${a_{1:k}}$ as follows:

\begin{align*}{\hat x_{k + 1|k}} = A{\hat x_{k|k - 1}} + K{\text{ }}\left( {{a_k} - {\text{ }}{C_a}{\text{ }}{{\hat x}_{k|k - 1}}} \right)\end{align*}

where $K$ is the Kalman gain for one-step-ahead prediction and ${\hat x_{i|j}}$ denotes the estimate of ${x_i}$ from neural observations up to time $j$, i.e. ${a_{1:j}}$. Given the extracted neural latent states, we can then predict neural activity in population B in the test data.

### 2.3. Smooth CroP-LDM to enable non-causal state smoothing

Given the noisy nature of the neural time-series, one may wish to forgo the interpretability benefits of causal filtering and instead perform non-causal smoothing of latent states for their more accurate estimation. Developing the smoothing capability will also enable fair comparisons with recent dynamical methods for cross-region interactions that are non-causal. However, doing so requires additional developments for model learning; for example, (Sani *et al* 2021) can only perform causal filtering but not non-causal smoothing when modeling behaviorally relevant neural dynamics, which is their goal. Thus, we further extend our CroP-LDM modeling approach to also support non-causal modeling (Sani and Shanechi 2025). To do so, the following additional steps are needed to extract the smoothed latent states. We start with the filtered estimate of our latent state:

\begin{align*}{\hat x_{k + 1|k + 1}} = {\text{ }}{\hat x_{k + 1|k}} + {K_f}{\text{ }}\left( {{a_{k + 1}} - {\text{ }}{C_a}{\text{ }}{{\hat x}_{k + 1|k}}} \right)\end{align*}

where ${K_f}$ is the Kalman gain matrix for the filtered estimate (Katayama 2006). Note that here the latest sample of activity from population A that has been used to estimate the state is ${a_{k + 1}}$, as opposed to ${a_k}$ in equation (3). This is indicated by the subscript of the estimated state being ${\hat x_{k + 1|k + 1}}$ versus ${\hat x_{k + 1|k}}$.

While the Kalman predictor of equation (3) depends only on learnable parameters, the Kalman filter of equation (4) depends on ${K_f}$, which is not uniquely learnable from the ${a_k}$ time series (see internal versus external descriptions in (Katayama 2006)). In our setup, however, we observe a second signal ${b_k}$. Therefore, of all the equivalent models, we can learn the ${K_f}$ that results in the best filtered estimation of ${b_k}$. To start, we get the following from equation (4) by multiplying both sides from the left with ${C_b}$:

\begin{align*}{\hat b_{k + 1|k + 1}} = {C_b}{\hat x_{k + 1|k}} + {C_b}{K_f}{ }\left( {{a_{k + 1}} - { }{C_a}{ }{{\hat x}_{k + 1|k}}} \right).\end{align*}

We then simplify and rearrange to get the following:

\begin{align*} {\hat b_{k + 1|k + 1}} - {\text{ }}{\hat b_{k + 1|k}} &amp; = {C_b}{K_f}{\text{ }}\left( {{a_{k + 1}} - {\text{ }}{C_a}{\text{ }}{{\hat x}_{k + 1|k}}} \right)\nonumber\\ &amp; = {C_b}{K_f}{ }\left( {{a_{k + 1}} - { }{{\hat a}_{k + 1|k}}} \right){ }{\text{}}.\end{align*}

Now, we fit a RRR with rank ${n_x}$ from $( {{a_{k + 1}} - {{\hat a}_{k + 1|k}}} )$ to $( {{b_{k + 1}} - {{\hat b}_{k + 1|k}}})$ to directly learn ${C_b}{K_f}$ in training data. Given this parameter, we can estimate the filtered target neural activity ${\hat b_{k + 1|k + 1}}$ using equation (5). The approach learns the Kalman filter parameter (i.e. ${C_b}{K_f}$) that can best update the estimate of ${b_k}$ after observing the latest sample of ${a_k}$. In other words, we can now predict ${b_k}$ using all past samples of ${a_k}$ (${a_1}$, …, ${a_{k - 1}}$) and the concurrent sample of ${a_k}$, i.e. we can get ${\hat b_{k + 1|k + 1}}$ instead of ${\hat b_{k + 1|k}}$.

We can next develop a smoothing procedure to non-causally infer ${b_k}$ using all past and future samples of ${a_k}$, i.e. using ${a_1}$, …, ${a_N}$ where $N$ is the total length of the data. The overall learning procedure for the smoothing models is as follows. First, in what we call the forward pass, we follow the approach explained above to learn the optimal Kalman filter using neural data from population A $\left( {{a_1},{\text{ }}{a_2}, \ldots {a_N}} \right)$ and population B $\left( {{b_1},{\text{ }}{b_2}, \ldots {b_N}} \right)$ in the training set. Once learned, this Kalman filter can be used to estimate the filtered target neural activity ${\hat b_{k + 1|k + 1}}$. Second, in what we call the backward pass, we follow the same approach above to learn a separate optimal Kalman filter on time reversed neural data from population A $\left( {{a_N},{\text{ }}{a_{N - 1}}, \ldots {a_1}} \right)$ and residual neural data from population B $( {{b_N} - {{\hat b}_{N|N}},{\text{ }}{b_{N - 1}} - {{\hat b}_{N - 1|N - 1}}, \ldots {b_1} - {{\hat b}_{1|1}}} )$ in the training set. Once learned, this backward pass Kalman filter can process the neural data from population A in the reversed direction, i.e. from future to past, and update all the estimates from the forward pass. Note that during the learning in the backward pass we use the *residual* target neural activity, which is the part of the target neural activity that the Kalman filter from the forward pass cannot predict. This is why the backward pass learns a model that can complement the predictions from the forward pass.

Once both models are learned in the training set as described above, they can be used for non-causal smoothing on the test set as follows. The forward pass allows us to estimate the filtered target neural activity ${\hat b_{k + 1|k + 1}}$ causally in time, using source data up to the concurrent sample, i.e. ${a_1}$, …, ${a_{k + 1}}$ with equation (4). The backward pass allows us to estimate any residual target neural activity that was not learned in the forward pass, by using additional data observed at $k + 2,{\text{ }}k + 3, \ldots ,{\text{ }}N$, which is not accessed in the forward pass. We sum the two sets of estimates from the forward and backward passes to get our final smoothed estimate of neural activity in population B, based on all past and future data from population A. Further theoretical derivations, proofs, and aspects of non-causal smoothing for general state-space models of two time series will be the topic of our other work (Sani and Shanechi 2025).

### 2.4. Baselines

#### 2.4.1. LDM

As a key baseline, we compare our results with LDM, which learns the parameters of the same model as CroP-LDM (equation (1)), but without prioritizing shared dynamics. As in CroP-LDM, LDM also describes a given neural population’s activity (e.g. population A) with a linear state-space model (equation (1), lines 1-2). However, it learns the parameters of this model without considering population B’s activity. This is because the objective of LDM is to explain the dynamics in population A. Specifically, during learning with subspace identification, LDM extracts the latent states by projecting future population activity in region A (${a_k}$) onto its own past. This is unlike CroP-LDM, which projects future neural activity from population B $\left( {{b_k}} \right)$ onto past neural activity from population A (${a_k}$). Once the model parameters are learned, in the training set, LDM infers the latent states using the associated Kalman filter and then fits a regression matrix ${C_b}$ from these latent states to Region B’s activity as in equation (1), line 3. The final learned model is identical to the CroP-LDM model (equation (1)). However, given the distinct way in which the model is learned, the dynamics captured in the latent states are not guaranteed to be shared across populations. Thus, the comparison of this baseline with CroP-LDM demonstrates the importance of prioritized learning of cross-population states.

#### 2.4.2. Mixed-loss LDM

A major capability offered by CroP-LDM compared with prior methods for cross-regional studies is the ability to prioritize the learning of cross-population dynamics, and separate them from within-population dynamics. Crop-LDM prioritizes the cross-population dynamics by making the sole objective of model learning to be the prediction of target activity using source activity. If predicting the source activity from its own past is also of interest, additional latent states can be appended to the model, but only via a second learning stage that optimizes a different self-prediction objective (appendix A)—though this is not our focus. The fact that cross-population dynamics are learned first with a distinct objective, allows these states to be prioritized in learning and also be dissociated from within-population dynamics to prevent the mixing of the two, which is a major challenge noted in prior studies (Stringer *et al* 2019).

As an ablation study, to isolate and show the benefit of prioritized learning, we develop a baseline with the exact same model structure as CroP-LDM (equation (1)), but this time learn it without the prioritized learning objective. In this baseline, we learn the same model as CroP-LDM, but this time maximize—using gradient descent—the joint log-likelihood of neural activity from both populations (${a_k},{\text{ }}{b_k}$ in equation (1)), which is similar to the objective used in many prior works (Glaser *et al* 2020, Gokcen *et al* 2022). We refer to this approach as Mixed-loss LDM, as it optimizes the combined likelihood of both the source and target neural activity and thus may include both cross-population and within-population dynamics in the learned model unlike CroP-LDM, which prioritizes the former. Note that after the model parameters are learned in the training set, inference of latent states for mixed-loss LDM in the test set is exactly the same as CroP-LDM: it uses a Kalman filter as in equation (3), but with different parameters that are directly learned during the numerical optimization of the mixed-loss LDM.

#### 2.4.3. Reduced rank regression

As another baseline to show the importance of modeling dynamics, we compare our CroP-LDM modeling approach with RRR (RRR; details in appendix B), which is a static method that has been used to model interactions across brain regions (Semedo *et al* 2019). RRR fits a linear model in which the coefficient matrix for the regression between regions is constrained to be low rank (Semedo *et al* 2019). In effect, RRR can be thought of as a regression that is composed of two linear mappings: (i) a mapping to a low dimensional latent variable with the specified dimension (i.e. the ‘rank’ in RRR), followed by (ii) a mapping from that latent variable to the target activity to be predicted. RRR is static and does not consider the temporal structure of the data.

#### 2.4.4. Baselines for non-causal modeling

We also compare our results with the state-of-the-art Delayed Latents Across Groups (DLAG; details in appendix C), which is a non-causal dynamic method that models the shared and within-region latent states as Gaussian processes (Gokcen *et al* 2022). As DLAG only supports non-causal inference of dynamics/states, our non-causal extension of CroP-LDM (which also uses future information from Region A to predict the activity in Region B, as explained in section 2.3) allows us to make the results comparable with DLAG.

### 2.5. Benefit of enabling inference both causally and non-causally and interpretation of directionality

CroP-LDM, LDM, and mixed-loss LDM have the capability to use only the past data from the source (population A) when inferring the latent states and predicting the target (population B), i.e. to perform causal filtering. DLAG on the other hand performs non-causal smoothing. The ability for causal filtering makes the results interpretable in the sense of information flow: information in population B that is predicted using population A must have appeared first in population A and then in population B. In contrast, if inference is done non-causally, information that appears later in population A could be used to ‘predict’ information that appears earlier in population B. Therefore, non-causal smoothing can lose interpretability in terms of the directionality of prediction/interaction between the two population. Nevertheless, non-causal smoothing has the upside of more accurate state estimation. CroP-LDM provides the capability to flexibly perform causal filtering or non-causal smoothing, whichever is desired. This is unlike prior methods that can only support one or the other.

### 2.6. Modeling interactions across and within regions

To model interactions across two regions, we take a neural population from each region and model their shared dynamics. To model interactions within a region, we take two non-overlapping populations within that region and again model their shared dynamics. While we could have also modeled the within-region activity by applying stage 2 of CroP-LDM (appendix A) on each region’s population activity, this approach risks the model learning trivial dynamics. Specifically, since stage 2’s learning objective is to use the activity of source region at each time-step to predict its own activity at the next time-step, it could simply learn to replicate the source activity from the previous time step rather than capturing true within-region dynamics. To avoid this interpretability issue, we modeled within-region dynamics by analyzing two non-overlapping sub-populations within a single region. Thus, in all cross-region and within-region analyses presented in Results, we always use CroP-LDM to model the cross-population dynamics of two non-overlapping populations, with the difference being only in how the two populations are selected.

For Monkey J, we randomly sample both the PMd and M1 electrodes to get four *non-overlapping* populations: ‘source’ PMd population, ‘target’ PMd population, ‘source’ M1 population, and ‘target’ M1 population, each of size 14. When the source and target populations are in the same region, we can model the shared dynamics within a single region.

For Monkey C, we focus on bilateral interactions. We thus first combine the electrodes in PMd and PMv of either hemisphere to generate a set of left and right premotor cortex (PMC) electrodes, where each set is of size 64. We then randomly sample both the Left PMC and Right PMC electrodes to get four *non-overlapping* populations: ‘source’ Left PMC population, ‘target’ Left PMC population, ‘source’ Right PMC population, and ‘target’ Right PMC population, each of size 14. For each recording session, we repeat this sampling procedure 10 times, resulting in a total of 70 datasets for Monkey J and 40 datasets for Monkey C. This repeated sampling increases statistical power and ensures that results are reflective of the interactions across typical populations sampled from the relevant regions, rather than being specific to any given population.

We evaluate how well activity in a given source population can predict activity in a given target population. To do so, for each method evaluated, we fit four models for each of the above four groups. In Monkey J, these are PMd to M1, M1 to PMd, PMd to PMd, and M1 to M1. For Monkey C, these are Left PMC to Right PMC, Right PMC to Left PMC, Left PMC to Left PMC, and Right PMC to Right PMC. These sets model interactions shared across populations in two regions or shared across populations within a region.

### 2.7. Cross-validated model evaluation

For each method in our analysis, we evaluate our models using five-fold cross-validation. We z-score the training and test data by subtracting the mean of the training data from both and normalizing both by the standard deviation of the training data. We use the cross-validated correlation coefficient (CC) between the true and predicted neural activity in the target population, averaged across neural dimensions, as our performance metric. We fit our CroP-LDM and LDM dynamical models using a horizon parameter of $i = 5$ in the subspace identification method (Van Overschee and De Moor 1996, Sani *et al* 2021).

### 2.8. Estimation of dimensionality for shared neural dynamics

We also quantify the dimensionality of the shared dynamics between two neural populations A and B. To do so, for the dynamical methods, we find the minimum number of latent states that sufficiently describe the neural activity in population A using the neural activity from population B. To do so, we use five-fold cross-validation to fit models with latent state dimensions ${n_x}$ ranging from 1 to 20. Then, we choose the optimal state dimension ${n_x}$ to be the smallest state for which the cross-validated CC, averaged across dimensions of the target region, is within one s.e.m. (standard error of mean) of peak performance over the latent state dimensions considered.

The dimensionality of the RRR model (i.e. the rank $m$ of the coefficient matrix) quantifies the number of dimensions in population A that are predictive of population B. For this method, we find the minimum $m$ such that the source population can sufficiently predict activity in the target population. To do so, we followed a similar procedure as described above, but fit models with dimension $m$ ranging from 1 to ${\mathrm{min}}({n_a},{\text{ }}{n_b})$, which are the number of neural observations in each region. In our analysis, for all source and target populations, we have ${n_a} = {\text{ }}{n_b} = 14$ given our construction in section 2.6.

### 2.9. Prediction of behavior from neural latent states

We also predict behavior from neural latent states as follows. Let ${r_k} \in {\mathbb{R}^{{n_r}}}$ represent the behavior measurements across ${n_r}$ dimensions of behavior at times $1 \ldots N$. We first use CroP-LDM on the training set consisting of source and target populations (i.e. ${a_k}$ and ${b_k}$) to learn the model parameters. We then use this learned model to estimate the latent states ${x_k} \in {\mathbb{R}^{{n_x}{\text{ }}}}$ in the training set using CroP-LDM’s inference procedure. Then, in the training set, we fit the projection matrix $L \in {\mathbb{R}^{{n_r} \times {n_x}{\text{ }}}}$ such that ${r_k} = L{x_k},$ using ordinary least squares as:

\begin{align*}{L_{{\mathrm{OLS}}}} = R{X^{\mathrm{T}}}{\left( {X{X^{\mathrm{T}}}} \right)^{ - 1}}.\end{align*}

Here columns of matrices $R \in {\mathbb{R}^{{n_r} \times N}}$ and $X \in {\mathbb{R}^{{n_x} \times N}}$ contain the behavioral measurements and predicted latent states over time. In the test set then, once the latent states are estimated with CroP-LDM’s inference procedure, we can simply apply ${L_{{\mathrm{OLS}}}}$ on them to estimate behavior.

### 2.10. Statistical tests

All statistical tests in this work were performed with the Wilcoxon signed-rank test. The Benjamini–Hochberg false discovery rate (FDR) correction was used to correct for multiple comparisons (Benjamini and Hochberg 1995).

## 3. Results

### 3.1. Validation in simulations: prioritized modeling accurately and efficiently learns cross-population dynamics

We first found the importance of the prioritized learning capability by comparing CroP-LDM with variants of the linear dynamical models trained without prioritized learning. We found that prioritized learning in CroP-LDM helped prevent confusing within-population dynamics for cross-population dynamics and allowed it to be more accurate and data-efficient for learning of cross-population dynamics. We did this in two analyses.

First, in simulations in which the ground-truth cross-population dynamics were known, we found that CroP-LDM more accurately learns these cross-population dynamics and also correctly prioritizes their learning over within-population dynamics. We simulated data from 50 random models with a total latent state dimension of ${n_x} = 8$ and a shared latent state dimension of ${n_1} = 4$. We then computed the error of learning the cross-population eigenvalues of the state transition matrix $A$ —i.e. the eigenvalues of the first ${n_1} \times {n_1}$ block of $A$ (appendix D)—, which characterize shared dynamics across populations. CroP-LDM prioritizes the learning of these cross-population eigenvalues, meaning it learns them first, in the first ${n_1}$ state dimensions. We swept the state dimension from 1 to 8 and learned a model for each simulation (figures 1(b) and (c)). CroP-LDM accurately learned all 4 of the cross-population eigenvalues with a minimum state dimension of 4 (figures 1(b) and (c), green), showing that it correctly learns the cross-population eigenvalues with priority over other eigenvalues. In contrast,

LDM, which does not prioritize the learning of cross-population dynamics, needed a much larger state dimension before it could identify the cross-population eigenvalues (figures 1(b) and (c), red). This is because LDM does not distinguish between the cross-population and within-population dynamics and thus may learn the within-population dynamics in the first few state dimensions, thus needing a larger latent state dimension to also learn all the shared dynamics. We then found that learning the same CroP-LDM model as in equation (1) but without the prioritized learning—i.e. mixed-loss LDM in section 2.4.2—was unable to learn the cross-population eigenvalues as accurately with the same minimum state dimension of 4 (figures 1(b) and (c), pink). Since the loss is a mix of both the source and target populations, mixed-loss LDM learned a mix of both the cross and within-population dynamics in the first few state dimensions (figure 1(b), pink). This shows that the prioritized learning enabled by the CroP-LDM objective was important for accurate learning of cross-population dynamics. Even when allowed to use higher latent state dimensions, the LDM variants without prioritization (LDM, mixed-loss LDM) were unable to reach the accuracy of CroP-LDM at latent state dimension of ${n_x} = 4$.

Second, we demonstrated that prioritization is critical to make the learning of cross-population dynamics data-efficient by repeating the modeling with different numbers of training samples. For a given number of training samples, LDM variants without prioritization exhibited much lower accuracy than CroP-LDM (figure 1(d)). Indeed, these methods required orders of magnitude more data to learn the cross-population eigenvalues as accurately as CroP-LDM. Finally, CroP-LDM learned the cross-population dynamics with an error that converged toward zero as more training samples were used (figure 1(c)).

Overall, these analyses showed that CroP-LDM enabled the best accuracy and data-efficiency for LDM of cross-population dynamics due to its prioritized learning procedure.

### 3.2. CroP-LDM more accurately models shared cross-population dynamics in the primate brain

Having shown the importance of CroP-LDM for dynamical modeling of cross-population interactions, we then used it to study cross- and within-region interactions in two NHPs. To study cross-region interactions, we modeled the dynamics shared between neural population from two distinct regions. For within-region interactions, we modeled the dynamics shared between two non-overlapping populations within the same region (section 2.6). Since, unlike a simulation, the ground-truth shared dynamics are unknown in these datasets, we assessed how well we identified the shared dynamics with CroP-LDM by predicting the target population’s activity from these shared states estimated purely from the source population’s activity. As main baselines, we compare with RRR and LDM because they also enable causally estimating the latent states for multiregional studies. Also, comparisons to RRR (static) and LDM (non-prioritized dynamic method) show the benefit of dynamical modeling and prioritized learning in CroP-LDM, respectively.

**Figure 2.**
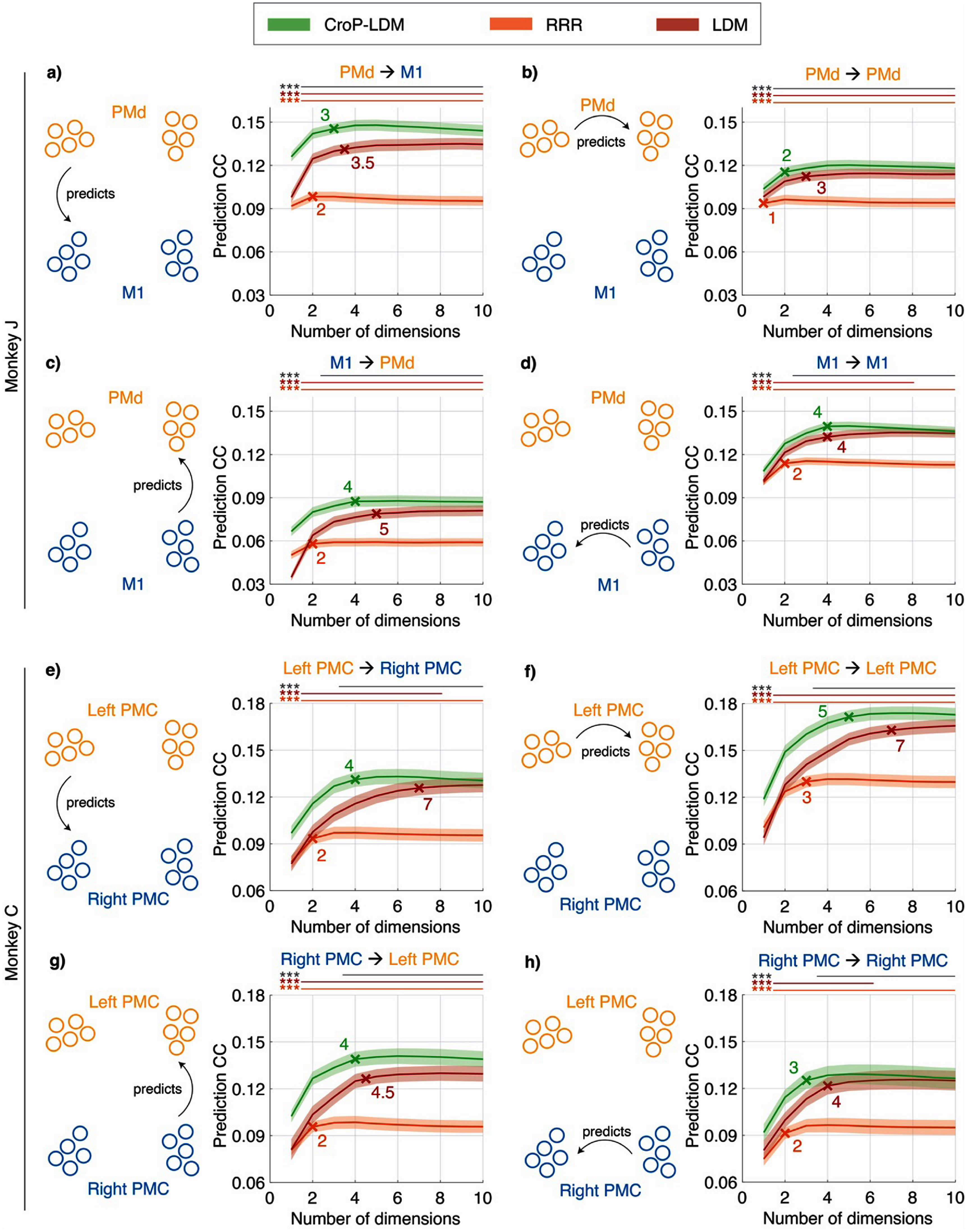
CroP-LDM more accurately predicts one population’s neural activity from another population’s past activity across state dimensions. (a) Comparing the cross-validated correlation coefficient (CC) of predicting one population’s activity from another across dimensions for CroP-LDM, RRR, and LDM, all of which perform the prediction causally in time, i.e. using only the past source activity. Solid lines show the CC averaged across the datasets and the shaded area indicates the s.e.m. ($n = 70$ datasets over $7$ sessions). The cross indicates the median dimension of the interaction at which performance saturates (see figures 5(a) and (b)). Across latent state dimensions, the statistical significance of a one-sided Wilcoxon signed-rank test, FDR corrected, is shown with a horizontal line and asterisks (gray: LDM vs. RRR; red: CroP-LDM vs. LDM; orange: CroP-LDM vs. RRR). Asterisks indicate significance of the comparison (*P < 0.05, **P < 0.005, ***P < 0.0005, and n.s. not significant). PMd significantly predicts M1 activity. (b) Same as (a) for PMd → PMd prediction. (c) Same as (a) for M1 → PMd prediction. (d) Same as (a) for M1 → M1 prediction. (e) Same as (a), but for monkey C ($n = 40$ datasets over $4$ sessions). Left PMC significantly predicts Right PMC activity. (f) Same as (e) for Left PMC → Left PMC prediction. (g) Same as (e) for Right PMC → Left PMC prediction. (h) Same as (e) for Right PMC → Right PMC prediction.

Across both datasets and all four region-pairs in each dataset, we found that CroP-LDM consistently outperformed both LDM and RRR in predicting the target activity, especially at low state dimensions (figure 2). Furthermore, dynamical modeling helped with performance as even LDM outperformed the static method RRR for almost all dimensions considered (figure 2). Indeed, even when RRR was allowed to use a higher dimensionality, both dynamical methods (LDM, CroP-LDM) still had a higher performance: CroP-LDM with ${n_x} = 1$ had stronger performance than RRR with any dimension, and a similar result held for LDM with ${n_x} = 2$. These results show the importance of dynamical modeling over static methods as the former can leverage the temporal structure of data to aggregate information over time.

**Figure A1.**
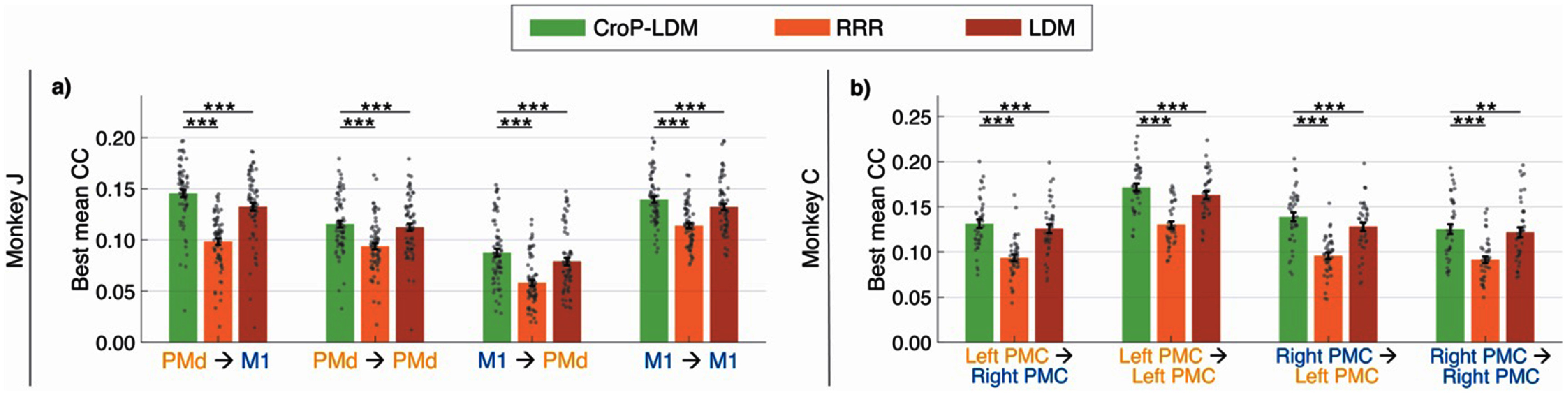
Across all region pairs, CroP-LDM outperforms RRR and LDM in terms of peak prediction of the target region from the source region. (a) The best CC for each method when using the optimal dimension (dimensions from figure 5(a)) for Monkey J ($n = 70$ datasets over $7$ sessions). Optimal dimension is defined as the minimum latent state dimension for which the method reaches within 1 s.e.m. of the peak prediction of the target region. Bars, whiskers, and asterisks as defined in figure 4(a). All data points are shown. (b) The best CC for each method when using the optimal dimension (dimensions from figure 5(b)) for Monkey C ($n = 40$ datasets over 4 sessions).

Finally, we found that CroP-LDM had significantly higher peak performance than LDM and RRR across datasets and region-pairs (figure A1), when the latent state was allowed to be as high as needed for each method. This result suggests that both the prioritized learning and dynamical modeling aspects offered by our method are important for learning shared neural dynamics. Given this result, we next used our method to quantify the strength of interactions between regions.

### 3.3. CroP-LDM can quantify dominant interactions between brain regions

We next used CroP-LDM to quantify the strength of interactions within and across populations of neurons. Critically, the peak prediction of one population from another (figure A1) is not directly comparable across different target populations because it may be confounded by the differences in noise level across populations. For instance, population A may be more predictable from population B than vice versa, not because the interaction pathway from B to A is stronger, but rather because A is generally easier to predict (e.g. less noisy). It is thus important to not only quantify the non-redundant information about a target that exists in the source, but also normalize that by how easy it is to predict the target in general.

Thus, to isolate and quantify how much population A predicts the activity in population B, we build two models with CroP-LDM: (1) a baseline model that quantifies how well past neural activity in B can predict future activity in B; (2) a model that quantifies how well past neural activity in both A and B can predict future activity in B. We again selected non-overlapping neural populations as described in section 2.6. As such, the difference in performance between these two models can isolate whether the past activity of population A predicts the present activity of population B, beyond what can be predicted by the past activity in population B itself. We now present these findings; note, a different quantification of these results based on a partial ${R^2}$ metric is discussed in section 3.4 and figure 4.

#### 3.3.1. Dataset 1: PMd and M1

**Figure 3.**
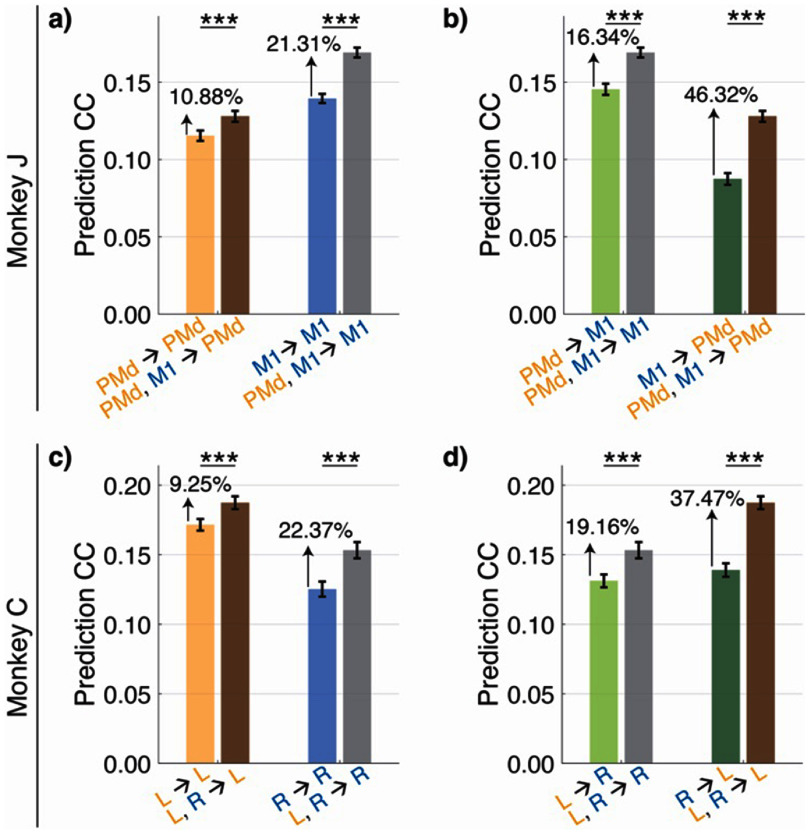
Using CroP-LDM to isolate regional effects. (a) Isolate additional contribution of M1 in across-region prediction (left). Isolate additional contribution of PMd in across-region prediction (right). Bars show the mean and whiskers show the s.e.m. For Monkey J, there are $n = 70$ datasets over $7$ sessions. Asterisks are as defined in figure 2. The notation on the *x*-axis ‘PMd, M1 → PMd’ means that the past activity of both PMd and M1 were used to predict the PMd activity and similarly for all other *x*-axis notations. (b) Isolate additional contribution of M1 in within-region prediction (left). Isolate additional contribution of PMd in within-region prediction (right). (c) Isolate additional contribution of Right PMC in across-region prediction (left). Isolate additional contribution of Left PMC in across-region prediction (right). For Monkey C, there are $n = 40$ datasets over $4$ sessions. (d) Isolate additional contribution of Right PMC in within-region prediction (left). Quantifying additional contribution of Left PMC in within-region prediction (right).

In Monkey J, we found that M1 helps in predicting PMd activity less than PMd helps in predicting M1 activity (11% increase vs. 21% increase; figure 3(a)). This result aligns with prior evidence that PMd sends input downstream to M1 (Civardi *et al* 2001, Koch *et al* 2006, 2007), and thus validates the utility of CroP-LDM for studying cross-region interactions. We also found that there is meaningful within-region interaction in both PMd and M1. Specifically, PMd was more driven by past PMd activity than by past M1, while M1 was driven by past PMd activity and past M1 activity (figure 3(b)). The results found by CroP-LDM are consistent with prior neurophysiological evidence, thus showing the biological consistency of its findings.

#### 3.3.2. Dataset 2: left and right PMC

In Monkey C, we found a strong within-area interaction for Left PMC. To see this, we note that prediction performance of neural activity in Left PMC went up by only 9% when we used past activity from Right PMC in addition to past activity from Left PMC (figure 3(c)). In contrast, prediction performance of neural activity in Left PMC went up much more, almost 37%, when we used past activity from Left PMC in addition to past activity from Right PMC (figure 3(d)). This result may be due to left PMC being in the hemisphere contralateral to the moving limb and thus being more modulated during movement. In comparison, the improvements to prediction of Right PMC were more similar regardless of whether Left PMC was added to Right PMC (22%, figure 3(c)) as predictor or vice versa (19%, figure 3(d)).

### 3.4. Quantifying non-redundant information across brain regions

As section 3.3 used raw predictive performances to quantify non-redundant information that exists in a source population about a target population, it had to do so by comparing two models: one that used the past target as predictor and one that used both the past source and past target as predictors. Here by ‘non-redundant’, we signify that the information does not exist in the past activity of the target population itself. As a more direct and interpretable measure of communication that does not need comparison of two models for each pair of regions, we next quantify the amount of *non-redundant* information about the target population by introducing a normalized measure of cross-population interactions based on partial ${R^2}$ (Bong *et al* 2020).

Partial ${R^2}{\text{ }}$ quantifies the improvement in predicting a quantity, when additional inputs are provided to the predictor. We compute the partial ${R^2}$ for predicting the target population’s activity when the past data from the source population is included in addition to the target population’s own past data (appendix E). This metric isolates the non-redundant contribution of the source population to the target population, while accounting for the target population’s inherent predictability. For example, this metric can allow us to quantify the contribution of past PMd to M1, after controlling for the autocorrelations in M1 that allow prediction of M1 activity using its own past.

#### 3.4.1. Dataset 1: PMd and M1

**Figure 4.**
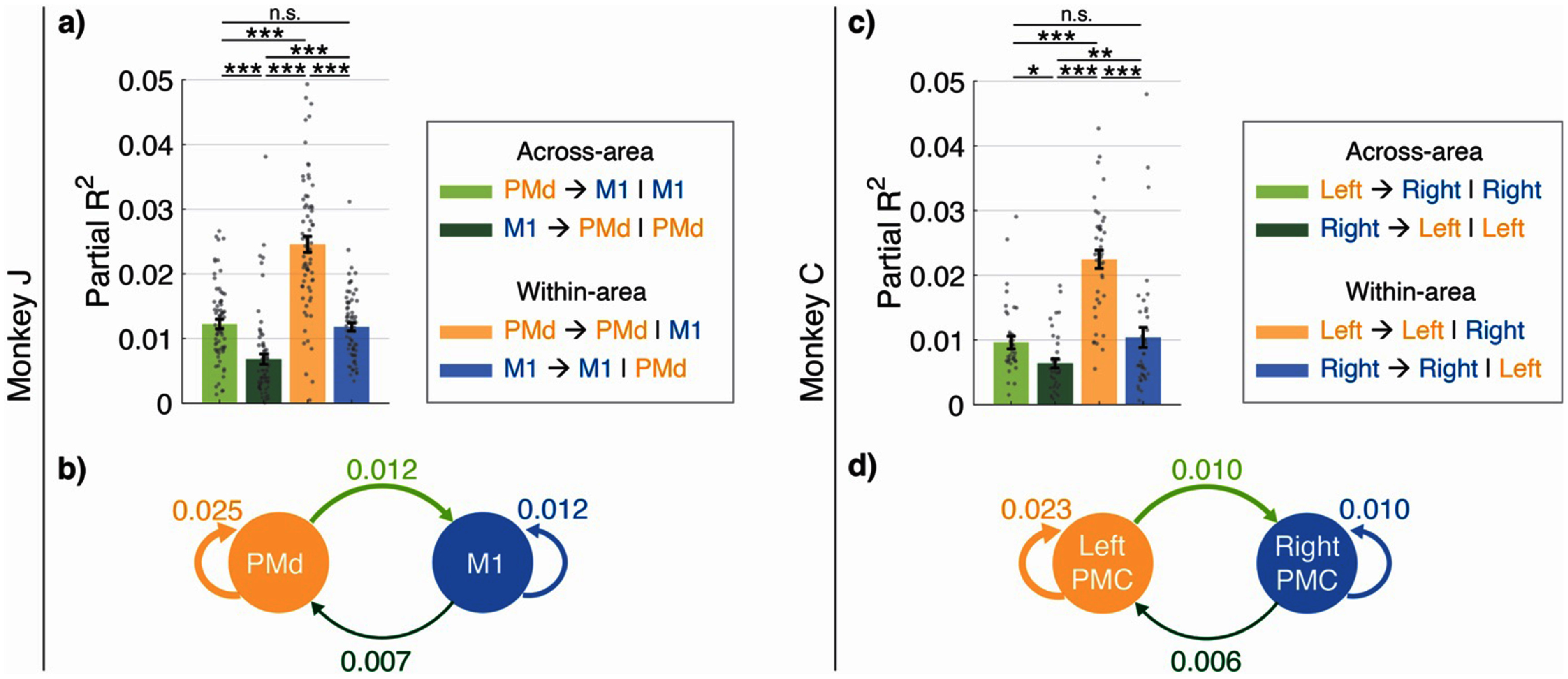
Using CroP-LDM to quantify and compare the strength of interactions between different neuronal populations. (a) The mean partial ${R^2}$ values attained for Monkey J by CroP-LDM when using the optimal state dimension for each cross- and within-region scenario. Bars show the mean and whiskers show the s.e.m. ($n = 70$ datasets over $7$ sessions). Asterisks are as defined in figure 2. All data points are shown. The legend ‘PMd → M1 | M1’ denotes the non-redundant information in PMd about M1 beyond that in M1 itself, as quantified by the partial ${R^2}$ measure. Other legend notations are also similar. (b) Given the mean partial ${R^2}$ values from a), we can visualize the dominant interactions within and across PMd and M1. The thickness of each line is proportional to the strength of that interaction pathway. (c) Same as (a) but for Monkey C ($n = 40$ datasets over $4$ sessions). Across and within Left PMC and Right PMC. (d) Same as (b) but for Monkey C across and within Left PMC and Right PMC.

Using CroP-LDM and as quantified by the appropriate partial ${R^2}$ metric, we found that the activity in PMd could predict the activity in M1, but the reverse relationship was much weaker (figures 4(a) and (b); first dataset). This finding is consistent with our comparisons in section 3.3, and aligns with the fact that the PMC is upstream of the primary motor cortex and sends input downstream to it (Civardi *et al* 2001, Koch *et al* 2006, 2007). This finding thus further validates the use of CroP-LDM to study cross-region interactions.

#### 3.4.2. Dataset 2: left and right PMC

In the second dataset, we found that the within-region interaction between neuronal populations in Left PMC (Left PMC → Left PMC), quantified by the partial ${R^2}$ metric, was significantly higher than the other three interactions (figures 4(c) and (d)), again consistent with our comparisons in section 3.3. Since the monkey was performing the task with its right hand, neural modulation is expected to be stronger in the Left PMC than in the Right PMC (Cisek *et al* 2003), thus again providing validation of CroP-LDM for studying cross-regional interactions. We also found that the across-region interactions (quantified by ${R^2}$) were similar in both directions, though Left PMC → Right PMC interaction was slightly higher than Right PMC → Left PMC. Prior work has suggested that the ipsilateral hemisphere may echo the behaviorally relevant activity of contralateral hemisphere with reduced strength or delayed output (Ganguly *et al* 2009, Fujiwara *et al* 2017). This may be why we observed a stronger interaction from the active contralateral hemisphere (left) toward the ipsilateral hemisphere (right), compared with the other direction.

### 3.5. Dimensionality of interaction: CroP-LDM can more accurately describe interactions using a low state dimension

We next used our method to study the dimensionality of the shared dynamics within and between regions and found that CroP-LDM more accurately identifies the shared state and its dimensionality compared with RRR and LDM. We define the dimension of the shared dynamics between two populations as the minimum dimension required to best predict the neural activity in one population given the neural activity from the other population (section 2.8). Overall, we found that across all region-pairs, CroP-LDM outperformed LDM and RRR in terms of the peak prediction accuracy of the target population from the source population (figure A1). Furthermore, we found that LDM led to an overestimation of the dimensionality while RRR led to an underestimation of this dimensionality given the below results.

**Figure 5.**
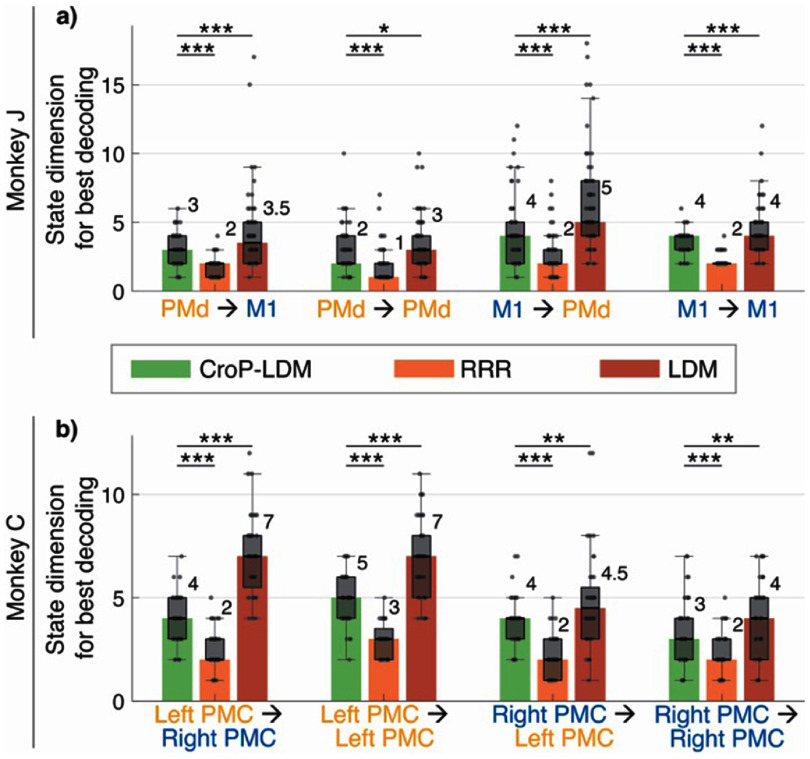
CroP-LDM identifies a low dimensional shared state across each region pair, while LDM overestimates and RRR underestimates the shared dimension. (a) The minimum state dimension that achieves within 1 s.e.m. of the best decoding for the four scenarios using CroP-LDM, RRR, and LDM. Bars show the median (also written in text). The boxes show the 25th and 75th percentiles, and the whiskers represent the minimum and maximum values, excluding outliers. Outliers are values more than 1.5 times the interquartile range away from the top and bottom of the box. Statistical significance is computed with one-sided Wilcoxon signed-rank test, FDR corrected ($n = 70$ datasets over $7$ sessions). Asterisks are as defined in figure 2. All data points are shown. The decoding accuracy associated with these dimensions is shown in figure A1, showing that while RRR reaches its peak prediction at a lower state dimension, that peak prediction is significantly worse than that of CroP-LDM. (b) Same as (a), but for Monkey C ($n = 40$ datasets over $4$ sessions).

Regarding LDM, (1) the dimensionality uncovered by CroP-LDM was lower than that found by LDM (figure 5) across all region-pairs and subjects and (2) LDM had a lower mean prediction performance at the dimension estimated by CroP-LDM (figure 2). At this dimension, CroP-LDM improved performance by up to 16% in Monkey J and 15% in Monkey C (figure 2). Furthermore, the prediction performance of CroP-LDM at this low dimension was higher than even a higher dimensional LDM (figures 2 and A1), indicating CroP-LDM better estimates the dimensionality while LDM overestimates it. While the gap between CroP-LDM and LDM narrowed for higher state dimensions (figure 2), peak performance of CroP-LDM remained significantly better than the peak performance of LDM across datasets and region-pairs (figure A1). Overall, LDM required more latent states to capture close to the same amount of the shared activity across regions as CroP-LDM does, resulting in an overestimation of the shared dimensionality compared with CroP-LDM.

Regarding RRR, while the dimensionality at which RRR performance peaked was lower than that of CroP-LDM (figure 5), this RRR peak performance was much lower than CroP-LDM performance, both at the low RRR dimension and at the CroP-LDM dimension (figure 2). Specifically, at the CroP-LDM dimension, CroP-LDM had an average performance improvement over RRR of up to 54% in Monkey J and 43% in Monkey C (figure 2). These results suggest that the RRR model underestimates the dimensionality of the shared interaction compared with CroP-LDM.

### 3.6. CroP-LDM’s uncovered states can predict behavior

We next found that the latent states extracted by Crop-LDM also contain behaviorally relevant information even though they are found unsupervised with respect to behavior. Further, the degree of this information was consistent with the roles of M1/PMd (dataset 1) and contralateral/ipsilateral PMC (dataset 2).

**Figure 6.**
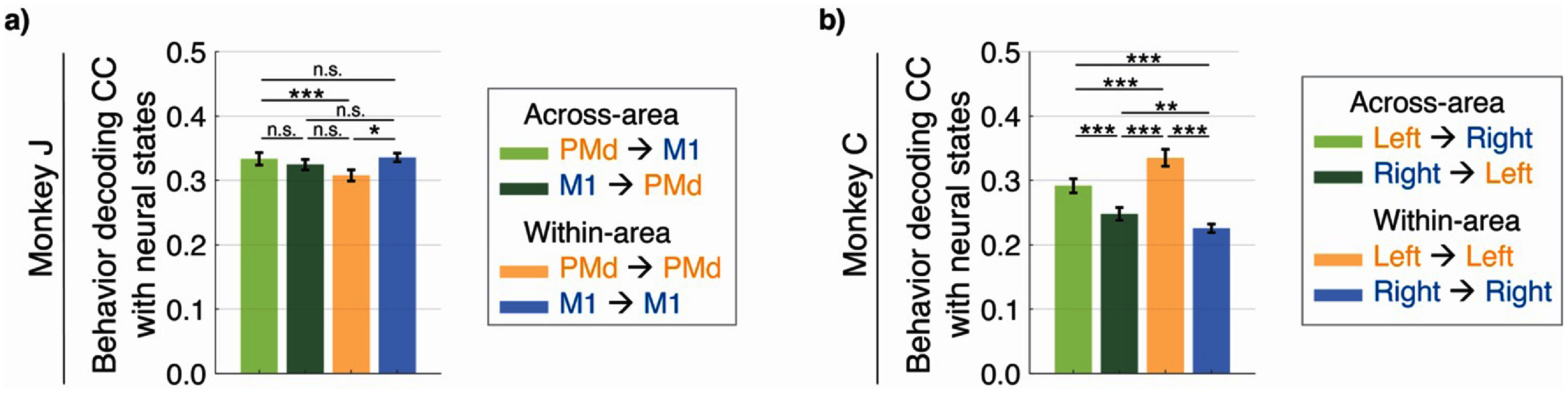
Average joint angle decoding accuracy for different latent states. (a) The peak average joint angle decoding accuracy using estimated neural latent states found by Crop-LDM. Behavior is decoded linearly from neural states, which are learned unsupervised with respect to behavior. The CC is averaged across the datasets. Bars show the mean and the whiskers show the s.e.m. ($n = 70$ datasets over $7$ sessions). Asterisks are as defined in figure 2. (b) Same as (a) but for Monkey C ($n = 40$ datasets over 4 sessions).

In each dataset, we used CroP-LDM to build 4 models and extract their associated latent states: shared within-region and shared across-region. Then, for each of these 4 sets of latent states, we learned a projection matrix from the latent states to behavior. We compared the peak behavior decoding accuracy for the 4 cases (figures 6(a) and (b)).

The shared feedforward PMd → M1 neural states decoded behavior with significantly higher accuracy than the PMd→PMd states and marginally higher than the feedback M1 → PMd states (figure 6(a)). This indicates that the shared information sent downstream from PMd to M1 is more behaviorally relevant than both the information that is shared within PMd and comparable or slightly more relevant than the information going in the feedback direction. M1 → M1 states achieved the highest behavior decoding of all the neural states, even marginally higher than the feedforward PMd → M1 neural states, suggesting that the computations taking place within the primary motor cortex were most directly related to behavior (kinematics).

In the second dataset, the latent states using left PMC activity as the source had the highest behavior decoding (figure 6(b)). This result aligns with the fact that the monkey is performing the behavioral task with its right arm, and therefore left/contralateral PMC activity is expected to be more predictive of behavior. The latent states using the ipsilateral hemisphere activity as the source could still decode behavior, albeit not as well as the contralateral hemisphere activity. This is consistent with prior studies showing that limb movement control is dominated by the contralateral hemisphere, but the ipsilateral primary motor and premotor cortices are still activated during unimanual limb movements (Tanji *et al* 1988, Aizawa *et al* 1990).

### 3.7. Non-causal modeling with CroP-LDM and baseline comparisons

In all prior analyses, we built models that causally predict the activity of a target population from the *past* activity of a source population. This ensures that predictive information appears in the source population before the ‘predicted’ activity appears in the target population. However, given the noisy nature of neural data, it may be of interest to also use the ‘future’ activity of the source population to enable more accurate though non-causal estimation of within- and cross- population latent states. For example, doing so can more accurately predict the target activity from source activity. Indeed, a recent state-of-the-art method for extracting cross-regional latent states has been non-causal (DLAG (Gokcen *et al* 2022)).

To enable such non-causal inferences, we extended the CroP-LDM learning to support optimal non-causal smoothing via a forward-backward procedure (section 2.3, Sani and Shanechi 2025). We refer to this version of CroP-LDM as smooth CroP-LDM. The added non-causal smoothing capability allowed us to compare smooth CroP-LDM to DLAG (Gokcen *et al* 2022). Note, DLAG does not explicitly learn a time evolution for the underlying latent states as is done in CroP-LDM. Rather, DLAG assumes that the activity evolves smoothly across time and models the latent states with independent Gaussian processes (appendix C).

**Figure 7.**
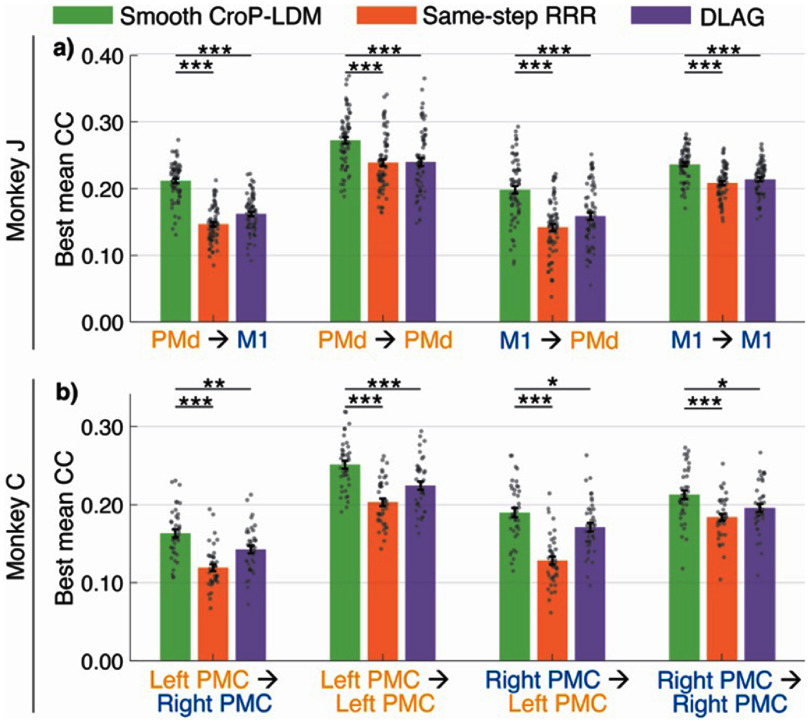
CroP-LDM with additional smoothing capability more accurately extracts shared dynamics compared to other baseline methods. (a) For Monkey J, the peak CC for smooth CroP-LDM, same-step RRR, and DLAG. The bars show the mean, and the whiskers show the s.e.m. ($n = 70$ datasets over 7 sessions). Asterisks are as defined in figure 2. State dimension for each method is selected as the smallest that reaches within 1 s.e.m. of the best prediction accuracy for that method. All data points are shown. (b) Same as (a) but for Monkey C ($n = 40$ datasets over 4 sessions). See figure A2 for low-dimensional regime comparisons.

**Figure A2.**
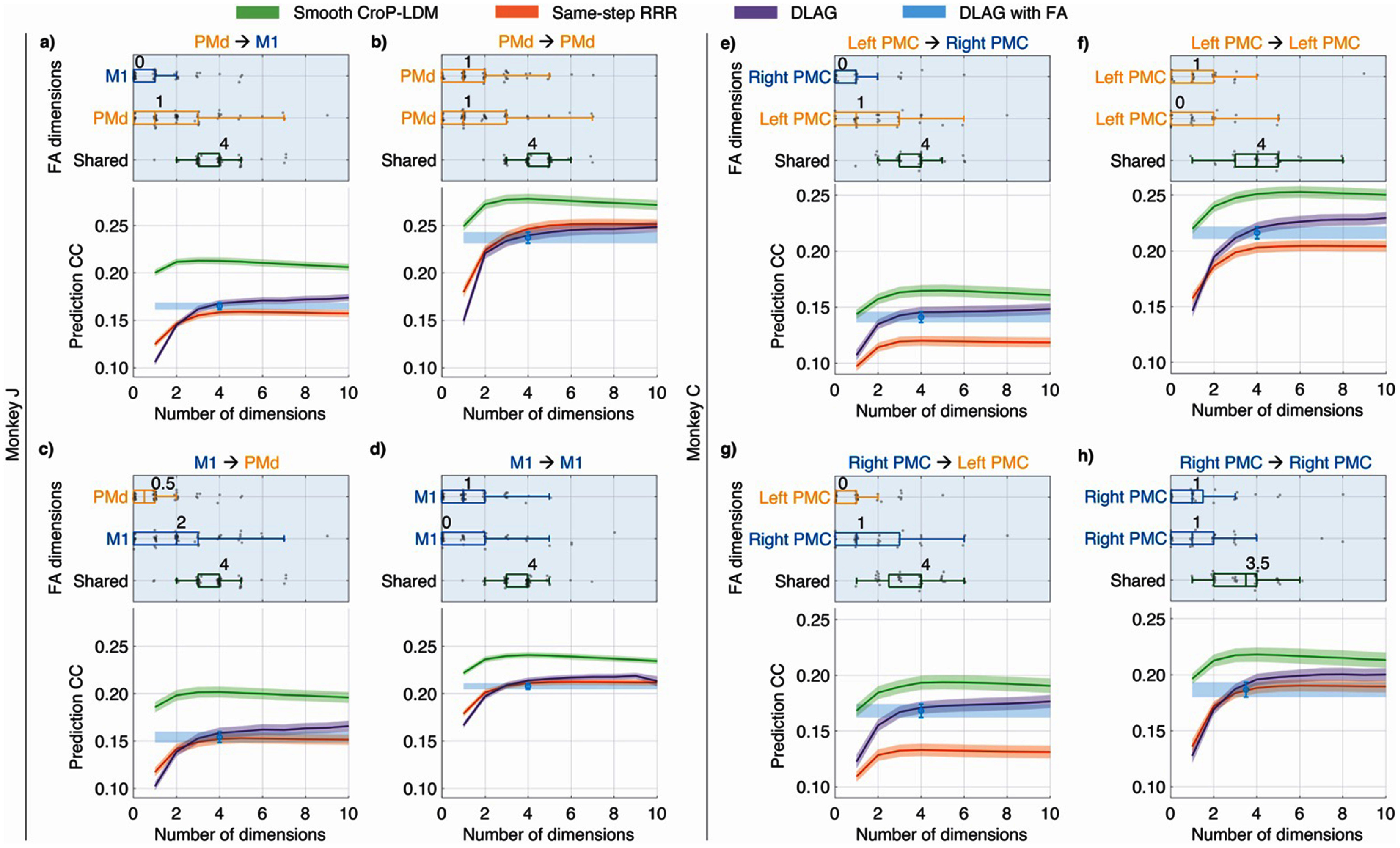
CroP-LDM with smoothing more accurately estimates one region’s population neural activity from another compared with alternative methods, across state dimensions. (a) Comparing the cross-validated CC of inferring one neural population’s activity from another neural population’s activity across dimensions for smooth CroP-LDM, same-step RRR, DLAG, and DLAG with Factor Analysis (FA). In the bottom panel, solid lines show the CC averaged across the datasets and the shaded area indicates the s.e.m. ($n = 70$ datasets over $7$ sessions). For DLAG, we assigned the total dimensionality for each region to be 10. For each DLAG model at a given shared dimensionality ${n_x}$, we set the within-region latent dimensionality for both regions to be $10 - {n_x}$. For DLAG with FA, we first applied factor analysis as a preprocessing step to each region independently to estimate the optimal total dimensionality for each region as is done in (Gokcen *et al* 2022). We then considered DLAG models where the number of shared and within-region latents summed to the optimal total dimensionalities. Among these candidate models, we chose the optimal model as the one that achieves the highest cross-region cross-validated CC. The blue point indicates the optimal CC for DLAG with FA averaged across the datasets, and the s.e.m. is shown as both error bars and a horizontal shaded area for ease of comparison. The *x*-coordinate of the point is the median of the shared state dimensionality across datasets. The top panel shaded in light blue shows the distribution of optimal dimensionalities chosen for the shared, within population A (i.e. PMd), and within population B (i.e. M1) latents. Bars and whiskers are defined as in figure 5(a). (b) Same as (a) for PMd → PMd prediction. (c) Same as (a) for M1 → PMd prediction. (d) Same as (a) for M1 → M1 prediction. (e) Same as (a), but for monkey C ($n = 40$ datasets over $4$ sessions) for Left PMC → Right PMC prediction. (f) Same as (e) for Left PMC → Left PMC prediction. (g) Same as (e) for Right PMC → Left PMC prediction. (h) Same as (e) for Right PMC → Right PMC prediction.

We compared the performance of smooth CroP-LDM with DLAG. We also compared with RRR modified to estimate the target activity at the same time-step as the source activity, termed same-step RRR (section 2.4.4) (figures 7(a) and (b)). Across datasets and region-pairs, smooth CroP-LDM outperformed both RRR and DLAG (figures 7(a) and (b)), particularly at low state dimensions, which is the dimensionality reduction regime (figure A2). These results suggest that provided with the same data as DLAG—i.e. both past and future activity in the source population—smooth CroP-LDM more accurately predicts the activity of the target population due to better learning the cross-population dynamics.

## 4. Discussion

We developed CroP-LDM, a framework for studying cross-population dynamics that prioritizes their learning to mitigate these dynamics being confounded or masked by within-population dynamics. In contrast to several prior static approaches for studying cross-regional interactions (e.g. RRR), CroP-LDM is a dynamic dimensionality reduction method that models the temporal structure in neural activity via a latent state-space model. This allows CroP-LDM to use the learned temporal autocorrelations for dimensionality reduction and for more accurate estimation of within- and cross-population latent states through temporal denoising (i.e. filtering/smoothing). Furthermore, unlike prior dynamic approaches (e.g. DLAG, LDM), CroP-LDM explicitly prioritizes the extraction of shared cross-population dynamics. We showed by doing so, CroP-LDM more accurately learns the cross-population dynamics than LDM and DLAG, as reflected in its better prediction of target activity from source activity. Finally, CroP-LDM provides the flexibility to extract the cross-population latent states causally (filtering) or non-causally (smoothing) in time. In comparison, DLAG provides non-causal estimation. This flexibility is important as causal filtering facilitates interpretability regarding the interaction by ensuring that only past source activity is used to predict future target activity, while non-causal smoothing enables more accurate latent state estimation when such interpretability is not desired.

### 4.1. Prioritization is important for accurate learning of cross-population dynamics in CroP-LDM

As a main contribution, we showed that prioritized learning is important for ensuring that the cross-population dynamics are learned accurately and are not confounded by within-population dynamics. To show this, we formulated a method called mixed-loss LDM that fits the same model as CroP-LDM, but instead of CroP-LDM’s prioritized learning objective, it learns this model by numerically optimizing the joint log-likelihood of source and target activity simultaneously, without prioritizing their shared dynamics. Indeed, prior works modeling interactions between brain regions have similarly used a mixed objective function that maximizes the likelihood of neural activity from both the source and target regions (Glaser *et al* 2020, Gokcen *et al* 2022). In simulations where the ground truth shared dynamics were known, we showed that mixed-loss LDM was unable to learn the shared eigenvalues as accurately as CroP-LDM. This is because the mixed loss benefits from learning both within- and cross-population dynamics unlike CroP-LDM, which is designed to prioritize learning the cross-population dynamics. Furthermore, CroP-LDM also better learned the cross-population dynamics compared with a (non-prioritized) LDM.

### 4.2. Comparisons to LDMs, RRR and DLAG

After establishing the strength of CroP-LDM among Linear dynamical system-based models (LDM and mixed-loss LDM as described above), we showed that across all state dimensions, CroP-LDM also outperforms RRR and DLAG, which have previously been used to model cross-regional interactions and are state-of-the-art methods (figures 7 and A2). These comparisons again highlight the importance of using dynamical modeling and prioritized learning of cross-regional dynamics.

In addition to accurate performance, another strength of CroP-LDM is its ability to extract the latent states either causally in time, using only past neural population data, or non-causally in time, using all available data. This is unlike DLAG, which only infers the cross-regional dynamics non-causally. CroP-LDM’s causal filtering can provide interpretability benefits while its non-causal smoothing achieves more accurate state estimation by leveraging both past and future data (compare CroP-LDM performance in figure 7 vs. figure A1). By extending CroP-LDM to support non-causal smoothing (Sani and Shanechi 2025), we also enabled comparisons with DLAG.

### Causal filtering and partial ${R^2}$to facilitate interpretability

4.3.

CroP-LDM can infer the dynamics/states both causally and non-causally in time while DLAG does so only non-causally in time. Despite their higher accuracy, non-causal inferences are not easily interpretable in terms of directional predictions because dynamics that appear later in the source region may be used by the non-causal model to predict those that appeared earlier in the target region. This can be inconsistent with the notion of ‘prediction’ and with the source and target definitions, and may hinder interpretability. Thus, in sections 3.3 and 3.4, where our goal was to interpret the strength of cross-population interactions, we opted to use CroP-LDM’s causal modeling. In this model, CroP-LDM extracts cross-population dynamics based only on past data from the source region, which ensures that results are interpretable as ‘predictions’. Furthermore, to compare interaction strengths across target regions, we used the partial ${R^2}$ metric, which quantifies the source region’s *non-redundant* contribution to the target region, while normalizing by the target region’s own predictability. Combining the causal modeling offered by CroP-LDM with this metric allowed us to fairly interpret and compare interaction strengths within and across regions.

### 4.4. Computational efficiency

The CroP-LDM model parameters are fit in a computationally efficient and non-iterative manner using standard matrix algebra and SVD computations. This is unlike various common dynamic methods, which fit model parameters iteratively using expectation maximization (Glaser *et al* 2020, Gokcen *et al* 2022). Unlike these, static methods, such as RRR, are often highly computationally efficient, but they lack the capacity to model the temporal structure of data. It may be possible to gain some insight towards time dependence while using a static method by fitting several models on time-shifted data (Semedo *et al* 2021); this approach, however, may not scale well as the number of time shifts increases. Meanwhile, CroP-LDM is a dynamical approach that explicitly models time dependence using a latent state-space model and is efficient at doing so.

### 4.5. CroP-LDM for studying cross-region and within-region dynamics

CroP-LDM can quantify the strength of interaction between populations of neurons (figure 4) by causally predicting the activity in one population from the past activity in the other and comparing their prediction strengths. In this study, we examined both cross-region and within-region interactions by modeling the shared dynamics between non-overlapping populations, either from distinct regions or within the same region, respectively. As an alternative method to modeling within-region interactions, we could have applied stage 2 of CroP-LDM (appendix A) to each region’s population. However, as stage 2’s objective is to predict a population’s activity at a given time from its own past activity, this alternative approach risks the model capturing trivial dynamics (e.g. replicating the previous time step of source activity as its current activity) rather than meaningful interactions within the region (section 2.6). We address this problem by modeling non-overlapping populations within a given region to learn non-trivial shared dynamics across distinct neurons in the same region. Nevertheless, this division into subpopulations results in fewer electrodes per population for modeling and is thus most appropriate in scenarios where the number of electrodes in a given region are not small.

### 4.6. CroP-LDM’s identified cross-population interaction strengths were biologically consistent

In the analysis of Monkey J, we were interested in understanding the communication pathway between PMd and M1 (figures 4(a) and (b)). Prior work has revealed reciprocal connections from the premotor areas of the same hemisphere to M1 (Civardi *et al* 2001, Dum and Strick 2002, 2006, Koch *et al* 2007). PMd has also been shown to play a strong role in the selection of actions (Koch *et al* 2006). We found that while M1 can predict PMd activity, this prediction is significantly weaker than that of M1 from PMd. This finding aligns with prior evidence that PMd is upstream of M1 and sends input down to M1. Moreover, the feedforward neural states extracted to capture the PMd → M1 interaction were more predictive of behavior than the feedback states (M1 → PMd) and the within PMd states (PMd → PMd); the within M1 states (M1 → M1) were most predictive of behavior in general. It has been shown that PMd is involved with the planning stages during motor tasks (Cisek and Kalaska 2005), while M1 is responsible for generating commands for movement (Dum and Strick 2002). This matches our finding that the within M1 neural states were most predictive of movement kinematics while the within PMd neural states were least predictive.

In Monkey C, we first found that neural predictability within Left PMC (contralateral) was significantly higher than the other three interactions (figures 4(c) and (d)). This aligns with prior work (Cisek *et al* 2003) showing stronger contralateral hemisphere activity during movement. Given that the task was performed with the monkey’s right hand, it is expected that neural modulation and thus predictability would be greatest within Left PMC. Furthermore, as expected, the latent states using Left PMC as the source achieved the highest behavior decoding (figure 6(b)). Next, we found a small but significant difference between the cross-region predictive abilities, with Left PMC → Right PMC being slightly stronger than Right PMC → Left PMC. Prior works have shown that the ipsilateral hemisphere is activated during unilateral limb movements and may weakly reflect the activity of the contralateral hemisphere (Tanji *et al* 1988, Aizawa *et al* 1990, Ganguly *et al* 2009, Hotson *et al* 2014, Bundy *et al* 2018). This may explain the stronger interaction from the contralateral hemisphere to the ipsilateral hemisphere, compared to the reverse direction.

### 4.7. All quantifications are still correlative

We emphasize that care must be taken when interpreting the interaction strengths found with CroP-LDM. Predictive ability as quantified by our method does not prove direct causality of the interaction between the two populations. Rather, it shows a correlation in the activity of the two populations. Moreover, even when we predict the target activity from the source activity causally in time (i.e. causal filtering), this does not imply causality in functional interaction but simply causality in time in terms of what data is used for prediction of target activity in a computational sense. So, while the source activity that is used to predict a target activity always appears before the target activity, the former may not be directly driving the latter. It may be the case, for example, that a common unobserved upstream region could be driving the activity seen in both source and target regions, but with different delays. It is important to interpret the results from this method within the context of the anatomical and functional connectivity of the brain regions analyzed. Ideally, any claims on causality should be backed up by perturbation experiments (Grosenick *et al* 2015, Bolus *et al* 2018, Shanechi 2019, Yang *et al* 2021b, Siddiqi *et al* 2022).

### 4.8. Future directions

Our CroP-LDM framework utilizes a linear state-space model. Linear models are widely used in neuroscience given their interpretability and flexibility. For example, one can examine the eigenvalues of the state transition matrix to study the oscillations and decay dynamics of neural populations (Abbaspourazad *et al* 2021, Song and Shanechi 2023, Vahidi *et al* 2024b). Linear state-space models are also computationally efficient and suited for real-time and closed-loop control applications, such as brain–computer interfaces (Gilja 2012, Orsborn *et al* 2012, Shenoy and Carmena 2014, Kao *et al* 2015, Shanechi *et al* 2017, Sani *et al* 2018, Yang *et al* 2018, 2019a, 2021b, Shanechi 2019, Degenhart *et al* 2020, Vyas *et al* 2020). However, nonlinear modeling may improve the expressivity of models of cross-regional interactions at the cost of interpretability. Indeed, recent theoretical work studying cross-regional communication between neural populations with biologically plausible synaptic dynamics has shown that when the receiving brain region is operating in a nonlinear regime, across-region communication may not be well measured by linear methods of communication (Gozel and Doiron 2024). Future nonlinear methods that have the capacity to model more complex shared dynamics and can quantify cross-population dynamics for within-region and cross-region analyses are important to develop and may lead to additional insights and also address potential model mismatch issues (Sani et al 2024).

While this work focused on modeling neural dynamics in the primate motor cortex during a movement task, the CroP-LDM framework is region-agnostic and is thus broadly applicable to other neural systems and behavioral tasks. Indeed, CroP-LDM can be readily applied to study interactions across any neural regions of interest, including motor, visual, cognitive, or sensory processing areas that have been the focus of prior cross-regional studies (Kaufman *et al* 2014, Arce-McShane *et al* 2016, Perich *et al* 2018, Rodu *et al* 2018, Ruff and Cohen 2019, Semedo *et al* 2019, Srinath *et al* 2021, Gokcen *et al* 2022). Moreover, CroP-LDM can be used to study neural activity during different behavioral paradigms, such as motor, sensory discrimination, and memory tasks (Buschman and Miller 2007, Salazar *et al* 2012, Mante *et al* 2013, Chen *et al* 2016, Wong *et al* 2016). Taken together, CroP-LDM provides a general framework that can be used across diverse neural systems and behavioral tasks in future studies to help in answering how interactions across brain regions enable sensory, cognitive, and motor functions—a fundamental question in neuroscience (Kohn *et al* 2020).

The latent state-space model in this work assumes stationary cross-population dynamics. These dynamics, however, may be nonstationary especially over longer periods of time, due to factors such as nonstationary task structures, learning, or plasticity. Prior work has developed adaptive state-space approaches including adaptive subspace identification methods that update model parameters in real time based on incoming neural data during inference, but without aiming to isolate cross-regional dynamics (Gilja *et al* 2012, Orsborn *et al* 2012, Shenoy and Carmena 2014, Shanechi *et al* 2016, Yang and Shanechi 2016, Hsieh and Shanechi, 2018, Degenhart *et al* 2020, Ahmadipour *et al* 2021, Yang *et al* 2019a, 2021a). An interesting future direction is to extend CroP-LDM to such nonstationary scenarios by developing adaptive subspace identification methods that can achieve prioritized modeling of shared neural dynamics across multiple regions or switching state-space models of shared dynamics, building upon prior studies that do not focus on isolating shared dynamics (Song *et al* 2022, Song and Shanechi 2023).This would enable the tracking of changes in cross-population dynamics in the presence of nonstationarities.

While our work analyzed spike counts as the neural modality, validation of our method on other modalities, such as intracranial electroencephalography, spiking-band power, and local field potentials, would be an interesting future direction (Chestek *et al* 2013, Chang 2015, Stavisky *et al* 2015, Bundy *et al* 2018, Hsieh *et al* 2018, Sani *et al* 2018, Anumanchipalli *et al* 2019, Wang and Shanechi 2019, 2019b, Nason *et al* 2020, Abbaspourazad *et al* 2021, Yang *et al* 2021b, Sadras *et al* 2023). Future work can also extend our method to allow for different data distributions, such as a point process distributions (Eden *et al* 2004, Coleman and Sarma 2010, Buesing *et al* 2012, Citi *et al* 2014, Sadras *et al* 2019) or multiscale distributions for multimodal data (Coleman *et al* 2011, Stavisky *et al* 2015, Hsieh *et al* 2018, 2019, Abbaspourazad *et al* 2021, Song *et al* 2022, Wang *et al* 2022, Ahmadipour *et al* 2024, Oganesian *et al* 2024, Sadras *et al* 2024). Extending the model to incorporate the effect of external inputs, such as sensory task stimuli, can also be another interesting direction (Susilaradeya *et al* 2019, Sauerbrei *et al* 2020, Vahidi *et al* 2024a, 2024b). Finally, the application of CroP-LDM to brain–computer interfaces that aim to decode and modulate neural activity using multi-regional recordings would be an interesting future direction (Citi *et al* 2008, Omar *et al* 2010, Shenoy and Carmena 2014, Shanechi *et al* 2016, Shanechi 2019, Nason *et al* 2020, Vyas *et al* 2020, Oganesian and Shanechi 2024, Hsieh and Shanechi 2025).

## Data Availability

The data that support the findings of this study are available upon reasonable request from the authors.

## Appendix A Extended model formulation for uncovering within-population dynamics

While not the focus of this work, it is possible to learn the within-population dynamics. For completeness, we outline the steps. When the learning of within-population dynamics is also of interest, our formulation can be extended to learn them in a second learning stage using projection-based subspace identification similar to (Sani *et al* 2021, Vahidi *et al* 2024b). In this extended formulation, we assume a block-structure for the latent states of the CroP-LDM model, as follows:

\begin{align*}\left\{ {\begin{array}{*{20}{c}} {\left[ {\begin{array}{*{20}{c}} {x_{k + 1}^{\left( 1 \right)}} \\ {x_{k + 1}^{\left( 2 \right)}} \end{array}} \right] = \left[ {\begin{array}{*{20}{c}} {{A_{11}}}&amp;0 \\ {{A_{21}}}&amp;{{A_{22}}} \end{array}} \right]\left[ {\begin{array}{*{20}{c}} {x_k^{\left( 1 \right)}} \\ {x_k^{\left( 2 \right)}} \end{array}} \right] + \left[ {\begin{array}{*{20}{c}} {w_k^{\left( 1 \right)}} \\ {w_k^{\left( 2 \right)}} \end{array}} \right]} \\[15pt] {{a_k} = \left[ {\begin{array}{*{20}{c}} {{C_{{a_1}}}}&amp;{{C_{{a_2}}}} \end{array}} \right]\left[ {\begin{array}{*{20}{c}} {x_k^{\left( 1 \right)}} \\ {x_k^{\left( 2 \right)}} \end{array}} \right] + {v_k}} \\ {{b_k} = \left[ {\begin{array}{*{20}{c}} {{C_{{b_1}}}}&amp;0 \end{array}} \right]\left[ {\begin{array}{*{20}{c}} {x_k^{\left( 1 \right)}} \\ {x_k^{\left( 2 \right)}} \end{array}} \right] + {\epsilon _k}} \end{array}} \right.,\,{x_k} = \left[ {\begin{array}{*{20}{c}} {x_k^{\left( 1 \right)}} \\ {x_k^{\left( 2 \right)}} \end{array}} \right].\end{align*}

In this extended formulation, $x_k^{\left( 1 \right)}$ represents the dynamics of population A that are shared with population B, and $x_k^{\left( 2 \right)}$ represents dynamics of population A beyond those that are shared with population B (i.e. are within population A). The block structure of zeros in the right-most block of ${C_b}$ indicate that only the shared dynamics $x_k^{\left( 1 \right)}$ drive population B. Also, the block structure in $A$ means that the evolution of the shared dynamics $x_k^{\left( 1 \right)}$ does not depend on the evolution of $x_k^{\left( 2 \right)}$.

As mentioned in Methods, in the first stage of learning, CroP-LDM extracts the shared states $x_k^{\left( 1 \right)}$ without them being confounded by any of the within-population A states $x_k^{\left( 2 \right)}$ (section 2.2.2). This explicit prioritization ensures that the dynamics learned as $x_k^{\left( 1 \right)}$ indeed reflect shared activity across populations A and B alone and are not mixed-up with within-population dynamics, which is a major challenge faced by prior methods for modeling cross-region dynamics (see introduction).

In the second optional stage of learning, it is possible to learn ${n_2}{\text{ }}$ additional within-population latent states by projecting residual future neural activity from population A onto past neural activity from population A. We did not apply the optional second stage in our neural data analysis (that is, we used ${n_x} = {\text{ }}{n_1})$ since we were interested only in extracting the states relevant to dynamics shared across populations. Indeed, even for within region analysis, we separated the region’s population into non-overlapping subpopulations and found their cross-population dynamics to study within-region dynamics (see Discussion section 4.5 and Methods section 2.6 for the reasons). Lastly, after the latent states are estimated via projection, the model parameters $A$, ${C_a}$, and ${C_b}{\text{ }}$ are estimated using least squares.

Of note, the block-structure in CroP-LDM’s formulation is inspired by (Sani *et al* 2021), which aims to find the behaviorally relevant neural dynamics in a single neural population. However, this prior work did not address the modeling of within and cross-population dynamics. Further, inference of latents in Sani *et al* at each time was limited to just using the past neural data (causal filtering) and could not incorporate future neural data (non-causal smoothing). Non-causal smoothing can be important for accurate discovery of cross-population interactions given the noisy nature of neural activity time-series in the two populations that are being modeled. Sani *et al* did not have to address this because in their problem, behavior is one of the time-series being modeled and is observed with much less noise than neural activity. Thus, as we show in section 2.3 (Sani and Shanechi 2025), we need to develop new inference methods to enable non-causal smoothing in addition to filtering.

## Appendix B RRR

Reduced-rank regression algorithm is a variant of linear regression in which the coefficient matrix for the interaction between population A and population B is constrained to be low rank. We can predict future neural activity of ${n_z}$ electrodes of population B with past activity from ${n_y}$ electrodes of population A using a linear model of the form $B = AX$. Here, $A \in {\mathbb{R}^{N - 1 \times {n_a}}}$ contains the activity of population A from time step $1$ to $N - 1$ and $B \in {\mathbb{R}^{N - 1{ } \times { }{n_b}}}$ contains the activity of population B from time step 2 to $N$. We shift the activity in the target population one time step ahead of activity in the source population when we are interested in comparing one-step-ahead prediction of neural activity in population B by RRR with that of the dynamic methods that perform one-step ahead prediction (i.e. CroP-LDM and LDM). The coefficient matrix $X \in {\mathbb{R}^{{n_a} \times {n_b}}}$ is found using ordinary least-squares via:

\begin{equation*}{X_{{\mathrm{OLS}}}} = {\left( {{A^{\mathrm{T}}}A} \right)^{ - 1}}{A^{\mathrm{T}}}B.\end{equation*}

To determine whether neural activity in population B can be predicted using a subspace of the source population, RRR imposes a rank constraint ${\mathrm{rank}}\left( X \right) = m$. The rank of the coefficient matrix quantifies the number of dimensions in population A that are predictive of population B. This defines the reduced-rank regression problem, which is solved using the singular value decomposition via:

\begin{equation*}{X_{{\mathrm{RRR}}}} = {X_{{\mathrm{OLS}}}}{V_m}V_m^{\mathrm{T}}.\end{equation*}

where ${V_m} \in {\mathbb{R}^{{n_b} \times m}}$ contains the top $m$ principal components of the optimal linear predictor ${B_{{\mathrm{OLS}}}} = A{X_{{\mathrm{OLS}}}} = U{{\Sigma }}{V^{\mathrm{T}}}$ with $U \in {\mathbb{R}^{N - 1 \times N - 1}},{{ \Sigma }} \in {\mathbb{R}^{N - 1 \times {n_b}}},{\text{ }}$ and $V \in {\mathbb{R}^{{n_b} \times {\text{ }}{n_b}}}$. To predict the neural activity in population B, we compute:

\begin{equation*}{\hat B_{{\mathrm{RRR}}}} = A{X_{{\mathrm{RRR}}}}.\end{equation*}

When comparing against non-causal versions of other methods (i.e. smooth CroP-LDM and DLAG), to enable a fair comparison, we adjust our setup to perform same step estimation instead of one-step-ahead prediction. To do so, we have $A \in {\mathbb{R}^{N \times {n_a}}}$ contain the activity of the population A from time step $1$ to $N$ and $B \in {\mathbb{R}^{N \times {n_b}}}$ contain the activity of population B from time step 1 to $N$. We emphasize that the RRR algorithm is not a dynamic method.

## Appendix C DLAG

We briefly review the DLAG algorithm which was introduced in (Gokcen *et al* 2022). DLAG is a multi-region extension of GPFA, with the added ability to model temporal delays between the regions. The model defines two types of latent variables, within-region $( {x_{i,j}^w})$ or shared across-region $( {x_{i,j}^a})$, where $i$ indexes the brain region and $j$ indexes the variable. Rather than explicitly modeling the temporal evolution of dynamics (via a linear dynamical system, for example), DLAG assumes that the latents evolve smoothly across time and models them as Gaussian processes (GP) with a squared-exponential covariance function. Each GP has a timescale parameter that controls its smoothness across time. The model defines a linear-Gaussian relationship between the observed neural activity ${y_{i,t}}$ and the latent variables via:

\begin{align*}{y_{i,t}} = C_i^ax_{i,:,t}^a + C_i^wx_{i,:,t}^w + {d_i} + { \epsilon_i}.\end{align*}

Here, model parameters $C_i^a$ and $C_i^w$ generate a linear combination of the latent variables, ${d_i}$ represents the mean firing rate of each neuron, and ${ \epsilon _i}$ is zero-mean Gaussian noise. The DLAG model parameters are estimated using expectation maximization.

We selected the number of within and shared latent variables in two ways. The first approach is described in detail in (Gokcen *et al* 2022) and summarized here briefly. Results from this approach are shown in figure A2 as ‘DLAG with Factor Analysis (FA)’. For each region, we used factor analysis to estimate its total dimensionality. Then, we fit a series of candidate DLAG models where the number of shared and within-region latents must sum to the FA total dimensionality for each region. Among these candidate models, we chose the optimal model as the one with the largest cross-validated CC on the held-out data between the true and predicted neural activity in the target region. In our second approach, we assigned each region to have a total latent dimensionality of 10. We then fit a series of candidate DLAG models, sweeping the shared dimensionality from 1 to 10 and constraining the shared and within-region latent dimensionality to sum to 10. This second approach allowed for a more direct performance trend comparison with the other methods (figure A2 ‘DLAG’; figure 7).

## Appendix D Numerical simulation details

We generated random parameters for numerical simulations as follows. For a chosen ${n_x}$ and ${n_1}$, we first generated eigenvalues of the state transition matrix $A$. To do this, we randomly drew complex conjugate pairs of points uniformly from the unit circle until we had a set of ${n_x}$ eigenvalues. We then randomly assigned ${n_1}$ of these as shared eigenvalues, while maintaining the property that these selected eigenvalues also form a set of complex conjugate pairs or real values.

We next generated ${C_a} \in {\mathbb{R}^{{n_a} \times {n_x}{\text{ }}}}$ by drawing each element from the standard normal distribution. Similarly, we generated ${C_b} \in {\mathbb{R}^{{n_b} \times {n_x}{\text{ }}}}$ by drawing each element from the standard normal distribution and setting the elements not associated with producing shared dynamics to $0$ (equation (8)).

Then, we generated matrices $Q$, $R$, and $S$ that determine the noise statistics. To do this, we first generated the matrix ${{\Omega }} \in {\mathbb{R}^{{n_x} + {n_a} \times {n_x} + {\text{ }}{n_a}{\text{ }}}}$ by drawing each element from the standard normal distribution. We then computed the positive-semi-definite matrix $M = {{\Omega }}{{{\Omega }}^{\mathrm{T}}}$. Next, we selected random scaling factors for the state and observation noise, ${a_1}$ and ${a_2}$, uniformly from $\left( { - 1,{\text{ }}1} \right)$. We then computed and extracted our noise matrices as follows:

\begin{align*}\left[ {\begin{array}{*{20}{l}} Q&amp;S \\ {{S^{\mathrm{T}}}}&amp;R \end{array}} \right] = \left[ {\begin{array}{*{20}{l}} {{{10}^{{a_1}}}{I_{{n_x}}}} \\ {{{10}^{{a_2}}}{I_{{n_a}}}} \end{array}} \right]M\left[ {\begin{array}{*{20}{l}} {{{10}^{{a_1}}}{I_{{n_x}}}}&amp;{{{10}^{{a_2}}}{I_{{n_a}}}} \end{array}} \right]\end{align*}

where ${I_n}$ is the identity matrix of size $n$.

Lastly, we generated the signal ${ \epsilon_k}$, which represents any independent neural activity in Region B not shared with Region A. To do this, we generated another random linear dynamical system with latent state dimension $n_x^{\prime}$ and corresponding parameters as described above. We then set the ratio of shared dynamics to independent dynamics in Region B. Specifically, we multiplied the rows of ${C_b}$ so that the ratio of the standard deviation of shared activity to the standard deviation of the independent activity equaled ${10^{{a_3}}}$, where ${a_3}$ was chosen uniformly from $\left( { - 1,{\text{ }}2} \right)$.

We used normalized eigenvalue error as our metric for evaluating the identification of shared eigenvalues. Given the ground truth shared eigenvalues and the identified shared eigenvalues, we found the closest pairing of the two sets over all possible pairings. When the total latent state dimension of the fitted model was smaller than the true shared latent dimension, we took the missing eigenvalues to be 0. We then computed the normalized eigenvalue error as

\begin{align*}\frac{{{{\left| {{v^{{\mathrm{(id)}}}} - {v^{{\mathrm{(true)}}}}} \right|}_2}}}{{{{\left| {{v^{{\mathrm{(true)}}}}} \right|}_2}}}\end{align*}

where ${v^{{\mathrm{(true)}}}}$ is a vector of the ground truth shared eigenvalues and ${v^{{\mathrm{(id)}}}}$ is a vector of the identified shared eigenvalues.

## Appendix E Partial ${R^2}$ metric

To compute the proportion of variation in population B explained by population A that cannot be explained by population B, we compute the following partial ${R^2}$ metric

\begin{align*}{\text{partial }}{R^2} = \frac{{{\mathrm{SSE}}\left( {\mathrm{B}} \right) - {\mathrm{SSE}}\left( {{\mathrm{A,B}}} \right)}}{{{\mathrm{SSE}}\left( {\mathrm{B}} \right)}}\end{align*}

where ${\mathrm{SSE}}\left( {\mathrm{B}} \right)$ denotes the sum of squares of the prediction error when only the past of population B is used for prediction, and ${\mathrm{SSE}}\left( {{\mathrm{A}},{\mathrm{B}}} \right)$ denotes the sum of squares of the prediction error when the past of both populations B and A are used for prediction (Bong *et al* 2020).
